# Glucagon-like peptide analogues for type 2 diabetes mellitus: systematic review and meta-analysis

**DOI:** 10.1186/1472-6823-10-20

**Published:** 2010-12-09

**Authors:** Deepson S Shyangdan, Pamela L Royle, Christine Clar, Pawana Sharma, Norman R Waugh

**Affiliations:** 1 Section of Population Health, Medical School Buildings, Foresterhill, University of Aberdeen, Aberdeen, AB25 2ZD

## Abstract

**Background:**

Glucagon-like peptide (GLP-1) analogues are a new class of drugs used in the treatment of type 2 diabetes. They are given by injection, and regulate glucose levels by stimulating glucose-dependent insulin secretion and biosynthesis, suppressing glucagon secretion, and delaying gastric emptying and promoting satiety. This systematic review aims to provide evidence on the clinical effectiveness of the GLP-1 agonists in patients not achieving satisfactory glycaemic control with one or more oral glucose lowering drugs.

**Methods:**

MEDLINE, EMBASE, the Cochrane Library and Web of Science were searched to find the relevant papers. We identified 28 randomised controlled trials comparing GLP-1 analogues with placebo, other glucose-lowering agents, or another GLP-1 analogue, in patients with type 2 diabetes with inadequate control on a single oral agent, or on dual therapy. Primary outcomes included HbA1c, weight change and adverse events.

**Results:**

Studies were mostly of short duration, usually 26 weeks. All GLP-1 agonists reduced HbA1c by about 1% compared to placebo. Exenatide twice daily and insulin gave similar reductions in HbA1c, but exenatide 2 mg once weekly and liraglutide 1.8 mg daily reduced it by 0.20% and 0.30% respectively more than glargine. Liraglutide 1.2 mg daily reduced HbA1c by 0.34% more than sitagliptin 100 mg daily. Exenatide and liraglutide gave similar improvements in HbA1c to sulphonylureas. Exenatide 2 mg weekly and liraglutide 1.8 mg daily reduced HbA1c by more than exenatide 10 μg twice daily and sitagliptin 100 mg daily. Exenatide 2 mg weekly reduced HbA1c by 0.3% more than pioglitazone 45 mg daily.

Exenatide and liraglutide resulted in greater weight loss (from 2.3 to 5.5 kg) than active comparators. This was not due simply to nausea. Hypoglycaemia was uncommon, except when combined with a sulphonylurea. The commonest adverse events with all GLP-1 agonists were initial nausea and vomiting. The GLP-1 agonists have some effect on beta-cell function, but this is not sustained after the drug is stopped.

**Conclusions:**

GLP-1 agonists are effective in improving glycaemic control and promoting weight loss.

## Background

### Type 2 diabetes mellitus

The primary aim of treatments for type 2 diabetes is to control blood glucose and reduce the development of diabetes-associated secondary complications[1]. Over time, persistently elevated levels of plasma glucose (hyperglycaemia) lead to various microvascular (retinopathy, nephropathy and neuropathy) and macrovascular (cardiovascular diseases) complications. The risk of developing such complications is strongly associated with the period of exposure to hyperglycaemia[2].

Initially people with type 2 diabetes should be advised on lifestyle changes (weight loss, increasing physical activity, diet) and offered ongoing patient education. If the lifestyle changes fail to control blood glucose then an oral glucose-lowering agent is given, usually metformin[3]. If metformin is contraindicated or not tolerated then sulphonylureas are considered. Type 2 diabetes is usually a progressive disease, with loss of beta cell capacity[4]. Once one oral glucose-lowering agent is insufficient to provide adequate glycaemic control a second is added (usually a sulphonylurea) to the initial metformin. Some oral glucose lowering agents have significant side effects, such as weight gain and hypoglycaemia, which might affect patient compliance and therefore glycaemic control. The American Diabetes Association (ADA) and the European Association for the Study of Diabetes (EASD) suggest that an HbA1c level of >7% indicates inadequate control[5].

At present, when people with type 2 diabetes have poor control on a combination of oral agents, the usual next step is to start insulin treatment[3], which often causes weight gain and hypoglycaemia, and often does not achieve good control[6].

Therefore existing treatments for these patients are unsatisfactory, and many patients do not have good glycaemic control, which will increase the risk of complications. New and better treatments are required.

### The glucagon-like peptide 1 analogues

GLP-1 analogues are a new class of glucose lowering drugs, given by injection, that mimic the action of an endogenous gastrointestinal hormone GLP-1, an incretin hormone that is released into the circulation in response to food. It regulates glucose levels by stimulating glucose dependent insulin secretion and biosynthesis, and by suppressing glucagon secretion, delaying gastric emptying and promoting satiety[7,8].

The action of the GLP-1 analogues is glucose-dependent i.e. the higher the plasma glucose level, the greater the effect of GLP-1 agonists on insulin secretion, with the greatest effect in hyperglycaemic conditions, and little or no effect when the blood glucose concentration is less than 3.6 mmol/L (65 mg/dL). This should reduce the occurrence of hypoglycaemia. The GLP-1 analogues have also been reported to produce weight loss in patients with type 2 diabetes[3,9-12].

There is some evidence from animal models that the GLP-1 analogues increases pancreatic islet beta-cell mass[13] and reduce beta-cell apoptosis[14,15]. If GLP-1 analogues do enhance beta-cell survival and stimulate beta-cell growth in individuals with type 2 diabetes, they may provide a means to preserve or restore functional beta-cell mass. This would be an important advance.

There are now at least six GLP-1 analogues: exenatide (Byetta, Lilly/Amylin) and liraglutide (Victoza, Novo Nordisk), that have reached the market, while albiglutide (GlaxoSmithKline) and taspoglutide (Ipsen and Roche) have been the subject of published trials. Others, including lixisenatide (sanofi-aventis) and LY2189265 (Lilly) are in the pipeline.

In the UK, the NICE guideline suggests that the GLP-1 analogues should be used as third line agents[3]. In the USA, they are often used as second-line.

There have been several good quality reviews of GLP-1 agonists [9-12] but most have included all trials, some of which are not relevant to clinical practice (e.g. patients who have not first failed metformin or a sulphonylurea or both), and none include trials published up to July 2010. This review aims to investigate the effectiveness of GLP-1 analogues in patients with type 2 diabetes mellitus who are not achieving satisfactory glycaemic control with one or more oral glucose lowering drugs.

## Methods

### Searching

The electronic databases MEDLINE (1996 to July 2010), EMBASE (1998 to July 2010), the Cochrane Library (July 2010) and Web of Science (1980 to July 2010) were searched for relevant papers.

The following Medline search strategy (Ovid) was adapted for use with the other databases:

1. exp Glucagon-Like Peptides/

2. (glucagon like peptide* or GLP-1).tw.

3. (exenatide or liraglutide or albiglutide or taspoglutide or lixisenatide).tw.

4. randomized controlled trial.pt.

5. (randomized or randomised).tw.

6. (1 or 2 or 3) and (4 or 5)

The websites of the Food and Drug Administration (FDA) and European Medicines Evaluation Agency (EMEA) were searched for information on efficacy and safety. Reference lists of relevant studies and reviews were also searched. We also searched Current Controlled Trials and ClinicalTrials.gov for ongoing trials. There were no language restrictions on the searches. One author and one company were contacted to provide or clarify standard deviations for two studies.

### Selection

Three authors (PR, DS, PS) scanned the titles and abstracts of every record retrieved. All potentially relevant articles were read as full text. Few differences in opinion existed, and these were resolved by a third party (NW).

Our inclusion criteria were randomised controlled trials that included patients with type 2 diabetes and were: published in full, had a minimum duration of 8 weeks, and compared a GLP-1 analogue with a placebo, insulin, an oral glucose lowering agent, or another GLP-1 analogue, in dual or triple therapy. These inclusion criteria were based on the comparisons which were considered to be relevant to clinical practice as suggested by the NICE guideline[3] and by the ADA/EASD joint statement[5].

Our exclusion criteria were trials where: GLP-1 analogues were used as monotherapy, patients were naïve to diabetes treatment, doses of GLP-1 analogues not used in clinical practice, arms differed in glucose-lowering co-medications, and at least two-thirds of participants had not been first tried on metformin prior to using GLP-1 analogues. The exception to this last criteria was for trials done in Japan, where there is low biguanide use and sulphonylureas are the most widely prescribed first-line treatment for type 2 diabetes[16,17], perhaps related to the low mean BMI (24.1 kg/m^2^) seen in Japanese people with type 2 diabetes[16]. In clinical practice, exenatide regimen is started with 5 µg twice daily and then increased after a month or so to 10 µg twice daily. The dose of liraglutide is less clear, with some trials suggesting starting with 0.6 mg, and then increasing in stages to 1.2 mg or 1.8 mg. So trials or arms with less than 1.2 mg daily, final dose, were excluded except for one trial[18] where liraglutide was used in the dose of 0.6 and 0.9 mg (standard available dose in Japan). For newer GLP-1 agonists, we only included dosages that were likely to be used in routine care - i.e. those with maximal effects while minimising adverse events.

### Validity assessment

The randomised controlled trials were assessed for quality using the criteria based on the Cochrane Collaboration's tool for assessing risk of bias[19]. The criteria were: method of randomisation, allocation concealment, blinding of participants, incomplete data addressed, free of selective reporting, groups comparable at baseline, and sample size calculation. Description of withdrawals or losses to follow up and reasons of withdrawals were systematically examined. Each study was quality assessed for the issues of incomplete outcome data or missing data by investigating drop-outs, losses to follow-up and withdrawals.

One of the three authors (CC, DS, PS) assessed the quality of each trial, and the assessment was checked by another author (CC, PR, DS). Any disagreements were resolved by consensus between the authors.

### Data abstraction

Two of the three authors (CC, DS, PS) independently extracted data using a standard data extraction form that was tested, piloted and modified for the current review. Data extraction was checked by a second author (CC, PR, DS). Relevant data on study population, intervention, study design and outcomes were pulled out from included studies. The few discrepancies found were resolved by consensus.

Primary outcome measures were: HbA1c, weight change and adverse effects, including hypoglycaemia. Other outcomes included BP (blood pressure), FPG (fasting blood glucose) and PPG (post-prandial glucose), plasma lipids, beta cell function, and health related quality of life.

### Quantitative data synthesis

Dichotomous data were expressed as the proportion of participants achieving the target HbA1c level of ≤7, and continuous data were expressed as weighted mean differences with 95% CIs. Analyses were done comparing different GLP-1 agonists against placebo or different active comparators.

Outcomes such as HbA1c, proportion of patients with HbAlc ≤7%, weight change and FPG were analysed using meta-analysis because all studies reported these outcomes using the same scale. The remaining outcomes were described qualitatively. For dichotomous data, event rates together with the sample size were used, whereas changes from baseline to end and standard deviations were computed for continuous data. Data were summarised with a random effects model using Review Manager 5. Heterogeneity was assessed using the I^2 ^test[19]. We regarded I^2 ^from 70% to 84% as substantial and 85% or more as highly significant. Due to the limited number of studies in each comparison, sensitivity analyses were not carried out. Relevant sensitivity analyses would have included analysis by study quality.

## Results

### Trial flow

**Figure 1:**
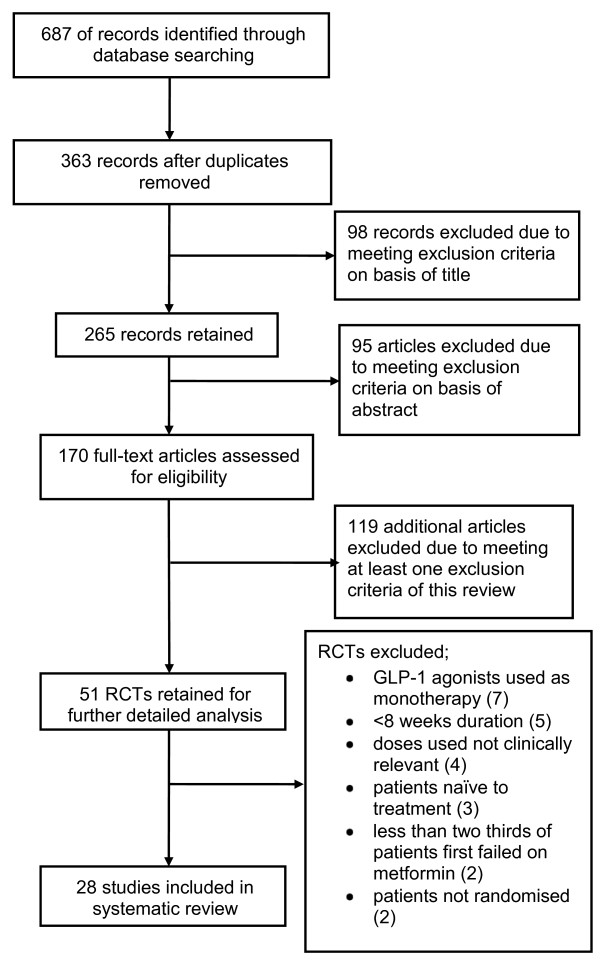
**Flow chart of search results**.

One hundred and seventy full text articles were assessed for eligibility and 142 papers were excluded. Twenty eight randomised controlled trials fulfilled the inclusion criteria and were included in the review (Figure 1). The main reasons for exclusion were: use of GLP-1 agonists as monotherapy, trials were <8 weeks duration, participants were not randomised, less than two-thirds of patients had been first tried on metformin, and the use of doses of GLP-1 agonists not relevant to those used in practice.

### Study characteristics

Of the twenty eight studies included, nineteen examined exenatide, seven liraglutide, one albiglutide, and two taspoglutide (one trial examined exenatide against liraglutide). The comparators included placebo, sulphonylurea, rosiglitazone, sitagliptin, pioglitazone and various forms of insulin. Characteristics of the included trials are shown in Table 1. They will be referred to in this section by the name of the first author and year of publication.

**Table 1:** Characteristics of the included studies.

Study and Country	Interventions	Characteristics of participants	Study duration	Outcomes measured
**ALBIGLUTIDE**				

Rosenstock 2009[23]; USA, Mexico, Chile, Dominican Republic	**1) Albiglutide 30 mg weekly** **2) Albiglutide 30 mg every two weeks** **3) Placebo** **All groups also received Met** *(The arms with other doses of albiglutide and exenatide excluded from this review)*	**number: **361 (31/32/51) **mean age: **54.0 to 55.5 years **gender: **45.1 to 74.2% female **HbA1c (%): **Albi Weekly 30 mg: 8.0, Placebo: 7.9, Albi every 2 weeks 30 mg: 8.0 **BMI (kg/m^2^): **Albi weekly 30 mg: 33.0, Albi every 2 weeks 30 mg: 31.2, Placebo: 31.8 **ethnicity: **Caucasian (43.8 to 71%) **diabetes duration: **4.9 years (3.9 to 5.2 years) **previous medication: **diet and exercise only: 29.0 to 34.4%, Met: 65.6 to 71.0%	16 weeks	**primary: **HbA1c **other: **FPG, fasting fructosamine, C-peptide, glucagon, insulin, lipid profiles, beta-cell function (homeostasis model), adverse events and safety analyses

**EXENATIDE**				

Apovian 2010[43]; US; 11 sites	**1) Exenatide 10 μg twice daily** **2) Placebo** **All groups also received intensive life style modifications and Met/Met + Su/Su**	**number: **196 (97/99) **mean age: **54.5 to 55.1 **gender: **62 to 63% female **HbA1c (%): **Exe 10 μg BID: 7.7; Placebo: 7.5 **BMI (kg/m^2^): **Exe 10 μg BID: 33.6; Placebo: 33.9 **ethnicity: **NR **diabetes duration: **5.3 to 5.7 years **previous medication: **Met; Met + Su; Su	24 weeks	**primary: **body weight **other: **HbA1c, 6-point SMBG profiles, waist circumference, HOMA-B, HOMA-S, fasting lipids, proportion of participants with weight loss >5% and 10%, SBP and DBP and subgroup analysis by oral agents

Bergenstal 2009[28]; USA	**1) Exenatide 10 μg twice daily** **2) BIAsp 30 twice daily** **3) BIAsp 30 once daily** **All groups also received Met + Su (glim)**	**number: **372 (124 in each comparison group) **mean age: **51.8 to 53.4 years **gender: **51.6 to 52.4% female **HbA1c (%): **Exe 10 μg BID: 10.2; BIAsp 30 OD: 10.1; BIAsp 30 BID: 10.3 **BMI (kg/m^2^): **Exe 10 μg BID: 34.2; BIAsp 30 OD: 33.7; BIAsp 30 BID: 33.5 **ethnicity: **59.7 to 67.7% White; 18.5% to 26.6% Black; 1.6 to 2.4% Asian **diabetes duration: **8.4 to 9.9 years **previous medication: **Met + Su	24 weeks	**primary: **HbA1c **other: **FPG; 8 point plasma glucose profiles changes in body weight; superiority and inferiority tested, adverse events

Bergenstal 2010[37]; USA, India, Mexico; 72 sites	**1) Exenatide 2 mg once weekly** **2) Sitagliptin 100 mg once daily** **3) Pioglitazone 45 mg once daily** **All groups also received Met + placebo**	**number: **491 (160/166/165) **mean age: **52 to 53 years **gender: **44 to 52% female **HbA1c (%): **Exe 2 mg QW: 8.6; Sita 100 mg OD: 8.5; Pio 45 mg OD: 8.5 **BMI (kg/m^2^): **Exe 2 mg QW: 32; Sita 100 mg OD: 32; Pio 45 mg OD: 32 **ethnicity: **30 to 39% White; 8 to 12% Black; 27 to 31% Hispanic; 23 to 25% Asian; 0 to 2% Native American; 1 to 2% other **diabetes duration: **5 to 6 years **previous medication: **Met	26 weeks	**primary: **HbA1c **other: **proportion of participants achieving HbA1c level ≤7% or ≤6.5%; FPG ≤7 mmol/l; 6-point SMBG; bodyweight; lipid profile; BP; cardiovascular risk markers; health-related outcomes; adverse events; hypoglycaemia; exenatide antibodies

Bunck 2009[29];	**1) Exenatide 10 μg twice daily** **2) Glargine 10 IU day, **titrated accordingly	**number: **69 (36/33) **mean age: **58.3 to 58.4 years	52 weeks	**primary: **Beta-cell function **other: **HbA1c; FPG; body weight; insulin sensitivity;

Netherlands, Finland, Sweden, USA	**Both groups also received Met**	**gender: **33.3 to 36.1% female **HbA1c (%): **Exe 10 μg BID: 7.6; Glar: 7.4 **BMI(kg/m^2^): **Exe 10 μg BID: 30.9; Glar: 30.1 **ethnicity: **NR **diabetes duration: **4.0 to 5.7 years **previous medication: **Met		safety; hypoglycaemia (BG <3.3 mmol/L)

Davies 2009 (HEELA)[30]; UK	**1) Exenatide 10 μg twice daily** **2) Glargine 10 IU/day, **titrated accordingly **Both groups also remained on previous therapy: **Met +/- Su +/- Tzd	**number: **235 (118/117) **mean age: **56.2 to 56.8 years **gender: **29.7 to 33.6% female **HbA1c (%): **Exe 10 μg BID: 8.65; Glar: 8.48 **BMI (kg/m^2^): **Exe 10 μg BID: 34.6; Glar: 33.7 **ethnicity: **NR **diabetes duration: **8.4 to 9.0 years **previous medication: **Met + Su: 42.3%; Met + Tzd: 13.7%; Su + Tzd: 2.6%; Met + Su + Tzd: 40.6%	26 weeks	**primary**: proportion of patients with an HbA1c level ≤7.4% and weight gain ≤1 kg i.e. composite outcome. **other**: body weight, waist circumference, FPG, serum lipids, BP, adverse events, hypoglycaemia

Davis 2007[31]; USA	**1) Exenatide 10 μg twice daily** **2) Insulin (remained on pre-study insulin regimen)** **Both groups also received Met+ Su**	**number: **51 (35/16) **mean age: **52 to 54 years **gender: **50 to 54% female **HbA1c (%): **Exe 10 μg BID: 8.0; Ins: 8.3 **BMI (kg/m^2^): **Exe 10 μg BID: 33; Ins: 35 **ethnicity: **NR **diabetes duration: **10.4 to 11.9 years **previous medication: **Met only: 43%; Su only: 8%; Met + Su: 49%; GLAR: 20%; NPH insulin: 2%; Ultralente: 2%; mixtures: 20%; multiple insulin therapies: 14%	16 weeks	**primary: **maintenance of glycaemic control, predefined as an HbA1c increase of <0.5% **other: **body weight; SMBG profiles; adverse events; hypoglycaemic events

DeFronzo 2005[42]; USA	**1) Exenatide 10 μg twice daily** **2) Placebo** **All groups also received Met** *(other dose of exenatide, 5 μg BID, excluded from this review)*	**number: **336 (113/113) **mean age: **52 to 54 years **gender: **39.8 to 40.7% female **HbA1c (%): **Exe 10 μg BID: 8.18; Placebo: 8.2 **BMI(kg/m^2^): **34 **ethnicity: **72.6 to 79.6% Caucasians; 8.8 to 13.3% Black; 8 to 10.6% Hispanic; 3.5% other **diabetes duration: **4.9 to 6.6 years **previous medication: Met**	30 weeks	**primary: **HbA1c **other: **safety, hypoglycaemia; anti-exenatide antibodies); FPG and PPG fasting proinsulin; lipids

DeFronzo 2010[22]; USA	**1) Exenatide 10 μg twice daily** **2) Rosiglitazone 4 mg twice daily** *(one arm receiving both exenatide and rosiglitazone was excluded from the review)* **All groups also received Met**	**number: **137 (45/45) **mean age: **56 years **gender: **49% female **HbA1c (%): **7.8 **BMI: 32.5** **diabetes duration: **3.7 to 4.7 years **ethnicity: **Caucasian, 61%, Hispanic 23%; African American 12%; Others 4% **previous medication: **Met ≥ 1500 mg/day	20 weeks	**primary: **measurement of glucose potentiated arginine stimulated incremental area under the curve during hyperglycaemic clamp test **other: **glucose AUC, HbA1c, glucose, insulin, C-peptide, lipids and body weight, adverse events, vital signs, haematology and chemistries.

Derosa 2010[27]; Italy	**1) Exenatide 10 μg twice daily** **2) Glibenclamide 5 mg, 3 times daily** **Both groups also received Met**	**number: **128 (63/65) **mean age: **57 to 56 years **gender: **52 to 49% female **HbA1c (%):**Exe 10 μg BID: 8.8; Glib: 8.9 **BMI (kg/m^2^): **Exe 10 μg BID: 28.7; Glib: 28.5 **ethnicity: **all white **diabetes duration: **NR **previous medication: **Met at mean dose of 1,500 ± 500 mg/day	12 months	**primary: **body weight, glycaemic control, β-cell function **other: **insulin resistance and inflammatory state parameters

Diamant 2010[34]; USA	**1) Exenatide 2 mg once a week** **2) Insulin glargine once daily**	**number: **456 (233/223) **mean age: **58 years **gender: **45 to 48% female **HbA1c (%): **Exe 2 mg QW: 8.3; Glar: 8.3 **BMI (kg/m^2^): **Exe 2 mg QW: 32; Glar: 32 **Ethnicity: **African American up to 1%, White 82 to 85%, Asian 6%, Hispanic 9 to 12% **diabetes duration: **7.8 to 8.0 years **previous medication: **Met: 70%; Met plus Su: 30%	26 weeks	**primary: **HbA1c **other: **proportion of participants reaching HbA1c <7.0% and 6.5%, FPG, self-monitored blood glucose, body weight, lipids, HOMA levels, health outcomes, adverse events, hypoglycaemia

Drucker 2008[41]; Canada/USA	**1) Exenatide 10 μg twice daily** **2) Exenatide 2 mg once weekly** **Both groups also remained on previous therapy: **diet/exercise or Met, Su, or Tzd as monotherapy or combination of any two.	**number: **303 (147/148) **mean age: **55 years **gender: **45 to 49% female **HbA1c (%): **Exe 2 mg QW: 8.3; Exe 10 μg BID: 8.3 **BMI (kg/m^2^): **Exe 2 mg QW: 35; Exe 10 μg BID: 35 **ethnicity: **73 to 83% White, 6 to 13% Black, 11 to 14% Hispanic, 0 to 1% Asian **diabetes duration: **6 to 7 years **previous medication: **Monotherapy: 43% to 46%, combination therapy: 36 to 39%; all Met: 69 to 77%, all Su: 37%, all Tzd: 15 to 17%; diet/exercise only: 14 to 16%	30 weeks	**primary: **HbA1c **other: **safety and tolerability, body weight, FPG, PPG, fasting lipids, fasting glucagon, BP, adverse events, patients who lost glucose control, hypoglycaemic episodes (symptoms and PG <3 mmol/l)

Gao 2009[45]; China, India, Korea, Taiwan	**1) Exenatide 10 μg twice daily** **2) Placebo** **Both groups also remained on previous therapy: **Met and/or Su	**number: **472 (238/234) **mean age: **54 to 55 years **gender: **52 to 59% female **HbA1c (%): **8.3 (in both groups) **BMI (kg/m^2^): **26.1 to 26.4 **ethnicity: **all Asian (Chinese 49.6 to 53%; Indian 20.3 to 21.4%; Korean 16.4 to 17.9%; Taiwanese 10.3 to 11.1%) **diabetes duration: **8 years **previous medication: **Met alone: 19.2 to 19.8%; Met and Su: 80.2 to 80.8%	16 weeks	**primary: **HbA1c **other: **body weight; hypoglycaemic events (symptoms and BG <3.3 mmol/l); FPG; PPG, adverse events, exenatide antibody levels

Gill 2010[26]; Canada and Netherland	**1) Exenatide 10 μg twice daily** **2) Placebo** **Both groups also remained on previous therapy: **Met and/or Tzd antihypertensives remained constant	**number: **54 (28/26) **mean age: **54 to 57 years **gender: **32 to 58% female **HbA1c (%): **7.1 to 7.5 **BMI (kg/m^2^): **29.5 to 30.1 **ethnicity: **Caucasian 86 to 96%; African 0 to 7%; East Asian 1%; Hispanic 0 to 1% **diabetes duration:** 6 to 7 years **previous medications:** Met±Tzd; antihypertensives	12 weeks	**primary: **24 hour heart rate (HR) **other: **HbA1c; body weight hourly; SBP; DBP; rate pressure product; hourly HR; daytime/night time HR; mean arterial pressure

Heine 2005[32]; 13 countries (Australia, 9 European countries, Brazil, Puerto Rico, USA)	**1) Exenatide 10 μg twice daily** **2) Glargine 10 IU/day, **titrated accordingly **Both groups also received Met and Su**	**number: **551 (282/267) **mean age: **58 to 59.8 years **gender: **43.4 to 45.0% female **HbA1c (%): **Exe 10 μg BID: 8.2; Glar: 8.3 **BMI: **Exe 10 μg BID: 31.4; Glar: 31.3 **ethnicity: **79.8 to 80.5% White; 0.7 to 1.1% Black; 0.7 to 1.8% Asian; 15 to 15.6% Hispanic; 2.1 to 2.6% other **diabetes duration: **9.2 to 9.9 years **previous medication: **Met+ Su	26 weeks	**primary: **HbA1c **other: **body weight, FPG, blood glucose, patient reported health outcome measures, adverse events, hypoglycaemia

Kadowaki 2009[20]; Japan	**1) Exenatide 10 μg twice daily** **2) Placebo** **Both groups also remained on previous medication: Su or Su +Met or Su +Tzd** *(Two other doses of exenatide, 2.5 μg BID and 5 μg BID were excluded from this review)*	**number: **153 (37/40) **mean age: **57.8 to 60.5 years **gender: **25 to 38% female **HbA1c (%): **Exe 10 μg BID: 7.9; Placebo: 8.1 **BMI(kg/m^2^): **Exe 10 μg BID: 26.1; Placebo: 25.8 **ethnicity: **presumably all Japanese **diabetes duration: **9.6 to 11.9 years **previous medication: **Su alone: 8.1 to 10%; Su + alpha-GI: 0 to 2.7%; Su + Met: 45 to 48.6%; Su + Met + alpha-GI: 18.9 to 22.5%; Su + Met + meglitinide derivative: 0 to 2.7%; Su + Tzd: 5.4 to 10%; Su + Tzd + alpha-GI: 12.5 to 13.5%	12 weeks	**primary: **HbA1c **other: **FPG; body weight; serum lipids, adverse events, hypoglycaemia (SMBG <3.9 mmol/l), amylase, antibodies to exenatide

Kendall 2005[21]; USA	**1) Exenatide 10 μg twice daily** **2) Placebo **(2 placebo arms combined) **Both groups also remained on Met + Su** *(other dose of exenatide, 5 μg BID, excluded from this review)*	**number: **733 (241/247) **mean age: **55 to 56 years **gender: **40.7 to 44.1% female **HbA1c (%): **Exe 10 μg BID: 8.5; Placebo: 8.5 **BMI: **Exe 10 μg BID: 34; Placebo: 34 **ethnicity: **66.4 to 69% White; 11.6 to 12.1% Black; 15.8 to 16.6% Hispanic; 1.6 to 2.9% Asian; 0.4 to 0.8% Native American; 1.6 to 2% other **diabetes duration: **8.7 to 9.4 years **previous medication: **Met and Su	30 weeks	**primary: **HbA1c **other: **FPG, PPG, body weight, lipids, adverse events, clinical laboratory tests, hypoglycaemia (BG <3.3 mmol/l)

Nauck 2007[33]; 13 countries	**1) Exenatide 10 μg twice daily** **2)BIAsp 30 twice daily** **Both groups also remained on Met + Su**	**number: **501 (253/248) **mean age: **58 to 59 years **gender: **47 to 51% female **HbA1c (%): **Exe 10 μg BID: 8.6; BIAsp 30: 8.6 **BMI (kg/m^2^): **Exe 10 μg BID: 30.6; BIAsp 30: 30.2 **ethnicity: **NR **diabetes duration: **9.8 to 10.0 years **previous medication: **Met and Su	52 weeks	**primary: **HbA1c **other: **FPG, PPG, SMBG profiles, beta-cell function, body weight, adverse events, anti-exenatide antibodies, hypoglycaemia (symptoms or BG <3.4 mmol/L)

Zinman 2007[67]; Canada, Spain, USA	**1) Exenatide 10 μg twice daily** **2) Placebo** **Both groups also remained on previous** **medication**: Tzd +/- Met	**number: **233 (121/112) **mean age: **55.6 to 56.6 years **gender: **42.9 to 46.3% female **HbA1c (%): **Exe: 7.9; Placebo: 7.9 **BMI (kg/m^2^): **Exe: 34; Placebo: 34 **ethnicity: **82.1 to 85.1% White; 14.9 to 17.9% other **diabetes duration: **7.3 to 8.2 years **previous medication: **Tzd alone: 19.6 to 23%; Tzd + Met: 76.9 to 80.4%	16 weeks	**primary: **HbA1c **other: **FPG, body weight, SMBG levels, HOMA levels, blood chemistry for safety monitoring; hypoglycaemia

**LIRAGLUTIDE**				

Kaku 2010[18]; Japan; 49 centres	**1) Liraglutide 0.6 mg once daily** **2) Liraglutide 0.9 mg once daily** **3) Placebo** **All groups also received Su**	**number: **264 (88/88/88) **mean age: **58.6 to 61.3 **gender: **33 to 40% female **HbA1c (%): **Lir 0.6 mg: 8.6; Lir 0.9 mg: 8.21; Placebo: 8.45 **BMI (kg/m^2^): **Lir 0.6 mg: 25.3; Lir 0.9 mg: 24.4; Placebo: 24.9 **ethnicity: **All Japanese **diabetes duration: **9.3 to 11.6 years **previous medication: **Su	24 weeks	**primary: **HbA1c **other: **7-point SMBG profiles, body weight, FPG, PPG, lipid profile and biomarkers for cardiovascular effects, proportions of subjects reaching HbA1c <7% or <6.5%

Marre 2009 (LEAD 1)[39]; 21 countries (mainly in Europe and Asia)	**1) Liraglutide 1.2 mg once daily** **2) Liraglutide 1.8 mg once daily** **3) Placebo** **4) Rosiglitazone 4 mg once daily** **All groups also received Su (Glim)** *(Lir 0.6 was excluded in this review)*	**number: **1041 (228/234/114/232) **mean age: **54.7 to 57.7 years **gender: **47 to 55% female **HbA1c (%): **Lir 1.2 mg: 8.5; Lir 1.8 mg: 8.5; Placebo: 8.4; Rosi: 8.4 **BMI (kg/m^2^): **Lir 1.2 mg: 29.8; Lir 1.8 mg: 30; Placebo: 30.3; Rosi: 29.4 **ethnicity: **NR **diabetes duration: **(median, 25^th^ and 75^th ^percentile) 6.5 to 6.7 years **previous medication: **monotherapy: 27/32%, combination: 68/73%	26 weeks	**primary: **HbA1c **other: **body weight, FPG, PPG, beta-cell function, BP; superiority of liraglutide to placebo and non-inferiority to rosiglitazone, hypoglycaemic episodes(PG <3.1 mmol/L), liraglutide antibodies, tolerability (gastrointestinal complaints), adverse events, biochemical and haematological parameters, calcitonin, vital signs, ECG

Nauck 2009 (LEAD 2)[36]; Multinational (21 countries)	**1) Liraglutide 1.2 mg once daily** **2) Liraglutide 1.8 mg once daily** **3) Glimepiride 4 mg once daily** **4) Placebo** **All groups also received Met** *(Lir 0.6 was excluded in this review)*	**number: **1091(240/242/242/121) **mean age: **56 to 57 years **gender: **40 to 46% female **HbA1c (%): **Lir 1.2 mg: 8.3, Lir 1.8 mg: 8.4, Su: 8.4, Placebo: 8.4 **BMI (kg/m^2^): **Lir 1.2 mg: 31.1, Lir 1.8 mg: 30.9, Su: 31.2, Placebo: 31.6 **ethnicity: **88 to 89% White, 2 to 4% Black, 7 to 9% Asian, 1 to 3% other **diabetes duration: **7 to 8 years **previous medication: **62 to 66% combination, 34 to 38% monotherapy (86 to 93% Met)	26 weeks	**primary: **HbA1c **other: **body weight, FPG, PPG, beta cell function, adverse events, biochemical and haematology measures, hypoglycaemic episodes (symptoms and PG <3.1 mmol/L), vital signs, ECG

Pratley 2010[38]; 11 European countries; USA and Canada; 158 office-based sites	**1) Liraglutide 1.2 mg once daily** **2) Liraglutide 1.8 mg once daily** **3) Sitagliptin 100 mg once daily** **All groups also received Met**	**number: **665 (225/221/219) **mean age: **55 to 55.9 **gender: **45 to 48% female **HbA1c (%): **Lir 1.2 mg: 8.4, Lir 1.8 mg: 8.4, Sita 100 mg: 8.5 **BMI (kg/m^2^): **Lir 1.2 mg: 32.6, Lir 1.8 mg: 33.1, Sita 100 mg: 32.6 **ethnicity: **82 to 91% White (15 to 17% Hispanic or latino), 5 to 10% Black, 1 to 3% Asian or Pacific Islander, 4 to 5% Other **diabetes duration: **6.0 to 6.4 years **previous medications: **Met	26 weeks	**primary: **HbA1c **other: **superiority and non-inferiority comparisons, proportion of participants reaching HbA1c targets of < 7% or ≤6.5%, FPG, PPG, body weight, β-cell function, fasting lipid profiles, cardiovascular markers, BP, HR, physical measures, treatment satisfaction, hypoglycaemia

Zinman 2009 (LEAD 4)[44]; USA and Canada	**1) Liraglutide 1.2 mg once daily** **2) Liraglutide 1.8 mg once daily** **3) Placebo** **All groups also received Met and Tzd (Rosi)**	**number: **533(178/178/177) **mean age: **55 years **gender: **38 to 49% female **HbA1c (%): **Lir 1.2 mg: 8.5, Lir 1.8 mg: 8.6, Placebo: 8.4 **BMI (kg/m^2^): **Lir 1.2 mg: 33.2, Lir 1.8 mg: 33.5, Placebo: 33.9 **ethnicity: **81 to 84% White, 10 to 15% Black, 13 to 16% Hispanic, 1 to 3% Asian, 3 to 4% Others **diabetes duration: **mean 9 years **previous medication: **16 to 18% monotherapy, 82 to 84% combination therapy	26 weeks	**primary: **HbA1c **other: **body weight, FPG, PPG, beta-cell function, BP, lipids, adverse events, biochemical and haematology measures, and hypoglycaemic episodes (PG <3.1 mmol/L), superiority of liraglutide tested, vital signs, ECG

Russell Jones 2005 (LEAD 5)[35]; Multinational (17 countries)	**1) Liraglutide 1.8 mg once daily** **2) Placebo** **3) Glargine **(avg. dose 24IU/day) **All groups also received Met and Su**	**number: **581(230/114/232) **mean age: **57.5 to 57.6 years **gender: **40 to 51% female **HbA1c (%): **Lir: 8.3, Placebo: 8.3, Glar: 8.2 **BMI (kg/m^2^): **Lir: 30.4, Placebo: 31.3, Glar: 30.3 **ethnicity: **NR **diabetes duration: **mean 9.2 to 9.7 years **previous medication: **5 to 6% monotherapy, 94 to 95% combination treatment)	26 weeks	**primary: **HbA1c **other: **weight, FPG, eight point plasma glucose profiles, beta-cell function, BP, adverse events, hypoglycaemic episodes

Buse 2009 (LEAD 6)[40]; 15 countries	**1) Liraglutide 1.8 mg once daily** **2) Exenatide 10 μg twice daily** **Both groups also remained on previous medications: Met +/- Su**	**number: **464(233/231) **mean age: **56 to 57 years **gender: **45 to 51% female **HbA1c: **Lir: 8.2, Exe: 8.1 **BMI: **Lir: 32.9, Exe: 32.9 **ethnicity: **91 to 93% White, <1 to 2% Asian, 5 to 6% Black, 1 to 2% other **diabetes duration: **mean 7.9 to 8.5 years **previous medication: **62 to 64% Met plus Su, 27% Met monotherapy, 9 to 10% Su monotherapy	26 weeks	**primary: **HbA1c **other: **FPG, body weight, SMBG profile, beta cell function, BP, lipid profiles, overall treatment satisfaction, adverse events, biochemical and haematological measures, hypoglycaemic episodes, vital signs, ECG

**TASPOGLUTIDE**				

Nauck 2009[24]; Germany and Switzerland	**1) Taspoglutide 10 mg once weekly** **2) Taspoglutide 20 mg once weekly** **3) Taspoglutide 20 mg once every two weeks** **4) Placebo** **All groups also received Met** *(For this review, the following groups were excluded: 5 mg weekly and 10 mg every 2 weeks)*	**number: **306 (49/50/49/49) **mean age: **56 years **gender: **39 to 64% female **HbA1c (%): **Placebo: 8.0, Tas 10 mg QW: 7.9, Tas 20 mg QW: 7.8, Tas 20 mg Q2W: 7.9 **BMI (kg/m^2^): **Placebo: 31.8, Tas 10 mg QW: 32.6, Tas 20 mg QW: 32.4, Tas 20 mg Q2W: 33.2 **ethnicity: **NR **diabetes duration: **mean 5 to 6 years **previous medication: **Met monotherapy (mean 1888 mg to 2019 mg)	8 weeks	**primary: **HbA1c **other: **FPG, body weight, fructosamine, C-peptide, fasting insulin, pro-insulin-to-insulin ratio, fasting glucagon, lipids, adverse events, clinical laboratory tests, local tolerance at the injection site, anti-taspoglutide antibodies, vital signs, ECG

Ratner 2010[25]; Australia, France, Germany, Mexico, Peru and USA; 27 sites	**1) Taspoglutide 20 mg once weekly** **2) Placebo** **All groups also received Met** *(For this review, the following groups were excluded: 20 mg once weekly titrated to 30 mg and 40 mg once weekly)*	**number: **133 (32/32) **mean age: **56 to 57 years **gender: **53 to 59% of female **HbA1c (%): **Tas 20 mg QW: 8.0, Placebo: 7.8 **BMI (kg/m^2^): **Tas 20 mg QW: 33.3, Placebo: 33.2 **ethnicity: **NR **diabetes duration: **6 to 7 years **previous medications: **Met	8 weeks + 4 weeks follow up	**primary: **GI tolerability **other: **HbA1c, FPG, body weight and pharmacokinetics parameters

Albi: Albiglutide; Exe: Exenatide; Lir: Liraglutide; Tas: Taspoglutide; Ins: Insulin; Glar: Insulin glargine; Tzd: Thiazolidinedione; Rosi: Rosiglitazone; Sita: Sitagliptin; Su: Sulphonylureas; Glim: Glimepiride; Met: Metformin; OD/QD: Once daily; BID: Twice daily; TID: Thrice daily; QW: Once weekly; HbA1c: Glycated haemoglobin; BMI: Body mass index; FPG: Fasting plasma glucose; PPG: Postprandial glucose; BG; Blood glucose; PG: Plasma glucose; BP: Blood pressure; SMBG: Self monitoring of blood glucose; HOMA: Homeostasis model assessment; ECG: Electrocardiogram; NR: Not reported; HEELA: Helping Evaluate Exenatide in patients with diabetes compared with Long-Acting insulin; LEAD: Liraglutide Effect and Action in Diabetes

The mean ages of the patients in the trials ranged from 51.8 to 61.3 years, mean baseline HbA1c ranged from 7.1% to 10.3%, and mean BMI from 25.0 to 35.0 kg/m^2^. The duration of the trials ranged from 8 to 52 weeks (the majority of the trials lasted 24 weeks or longer and two studies lasted 52 weeks). The mean number of participants in trials was 383 (range 51 to 1091); however, eight studies had fewer than 50 participants.

All studies, except Derosa 2010, had industry affiliation or sponsorship from the manufacturer of the trial drug. Buse 2009 (the trial of liraglutide versus exenatide) was sponsored by Novo Nordisk, the manufacturer of liraglutide.

In some studies there were some uncertainties or inequalities regarding previous or concomitant anti-diabetic treatment. Patients in Marre 2009 (LEAD-1) were assumed to have been on previous metformin therapy, but this was not reported in the study. In Davis 2007, there were substantial imbalances in concomitant treatment i.e. 12% of patients in the exenatide group being on previous sulphonylurea only, and none in the insulin group; also, insulin therapy in the comparison group was not optimised. In Bergenstal 2009, sulphonylurea was discontinued in the BIAsp 30 twice daily group to reduce the risk of hypoglycaemia, but not in the other groups where patients received both a sulphonylurea and metformin.

The primary outcome measure in most trials was change in HbA1c value from baseline to the end of the study. In all trials except one, more than 70% of the patients were on metformin. In Kaku 2010 all the participants were primarily on sulphonylureas (the standard first line oral antidiabetic drug in Japan)[16].

#### Groups excluded from this review

We excluded some arms of the included trials. In two trials[20,21] the group of patients taking non clinically relevant dose of exenatide were excluded. In DeFronzo 2010[22] the participants taking both exenatide and rosiglitazone were excluded. Some doses of albiglutide[23] and taspoglutide[24] used in dose ranging trials were excluded, as, based on the results or the author's conclusions, they seemed unlikely to be used in clinical practice. In Rosenstock 2009[23], a group receiving exenatide was excluded, as all participants in this group also received metformin, whereas only a proportion of the patients in the other groups did. Ratner 2010[25] compared three doses of taspoglutide (20 mg once weekly escalated to 20 mg, 30 mg and 40 mg once weekly). In this review, we only considered 20 mg once weekly escalated to 20 mg once weekly.

### Study quality

Studies were mainly of moderate to high quality, with most studies fulfilling five to seven of the seven quality criteria. Note that blinding was not practical in some trials. Table 2 shows the quality of the included trials. Four studies only fulfilled four of the criteria, while ten fulfilled five criteria, seven fulfilled six criteria, and seven fulfilled seven criteria. As shown in Table 2 many studies had one or more arms with losses to follow-up of 20% or more.

**Table 2:** Quality of included trials.

Study	Adequate sequence generation	Allocation concealment	Blinding	Incomplete outcome data addressed	Percentage who completed the trial	Free of selective reporting	Groups comparable at baseline	Sample size calculation
Rosenstock 2009[23]	Unclear	Unclear	Yes, Double blind	Yes	Albi 30 mg QW/Albi 30 mg every two weeks/P: 71.0/75.0/78.4	Yes	Yes	Yes

Apovian 2010[43]	Yes	Yes	Yes, Double blind	Yes	Exe/P: 72.2/72.7	Yes	Yes	Yes

Bergenstal 2009[28]	Yes	Yes	No, Open label	Yes	Exe/BIAsp QD/BIAsp BID: 70.2/83.9/80.6	Yes	Yes	Yes

Bergenstal 2010[37]	Yes	Yes	Yes, Double blind	Yes	Exe/Sita/Pio: 79.4/86.7/79.4	Yes	Yes	Yes

Bunck 2009[29]	Yes	Unclear	Unclear	Yes	Exe/Glar: 83.3/90.9	Yes	Yes	Yes

Davies 2009 (HEELA)[30]	Yes	Unclear	No, Open label	Yes	Exe/Glar: 83.9/88.9	Yes	Yes	Yes

Davis 2007[31]	Unclear	Unclear	No, Open label	Yes	Exe/Ins: 57.6/93.8	Yes	Yes	Yes

DeFronzo 2005[42]	Unclear	Unclear	Yes, Triple blind	Yes	Exe/P: 82.3/78.8	Yes	Yes	Yes

DeFronzo 2010[22]	Yes	Unclear	No, Open label	Yes	Exe/Rosi: 73.3/75.6	Yes	Yes	No

Derosa 2010[27]	Yes	Yes	No, Single blind	Yes	Exe/Glib: 93.7/87.7	Yes	Yes	No

Diamant 2010[34]	Yes	Yes	No, Open label	Yes	Exe/Glar: 89.7/93.7	Yes	Yes	Yes

Drucker 2008[41]	Unclear	Unclear	No, Open label	Yes	Exe/Exe lar: 88.4/86.5	Yes	Yes	Yes

Gao 2009[45]	Yes	Yes	Yes, Double blind	Yes	Exe/P: 81.1/88.9	Yes	Yes	Yes

Gill 2010[26]	Unclear	Unclear	Yes, Double blind	Yes	Exe/P: 78.6/88.5	Yes	Yes	Yes

Heine 2005[32]	Yes	Yes	No, Open label	Yes	Exe/Glar: 80.9/90.6	Yes	Yes	Yes

Kadowaki 2009[20]	Unclear	Unclear	Yes, Double blind	Yes	Exe/P: 83.8/97.5	Yes	Yes	Yes

Kendall 2005[21]	Unclear	Unclear	Yes, Double blind	Yes	Exe/P: 82.6/76.1	Yes	Yes	Yes

Nauck 2007[33]	Yes	Yes	Unclear	Yes	Exe/BIAsp 30 BID: 78.7/89.9	Yes	Yes	Yes

Zinman 2007[67]	Yes	Yes	Yes, Double blind	Yes	Exe/P: 71.1/85.7	Yes	Yes	Yes

Kaku 2010[18]	Unclear	Unclear	Yes, Double blind	Yes	Lir 0.6/Lir 0.9/P: 94.3/95.5/84.1	Yes	Yes	Unclear

Pratley 2010[38]	Yes	Yes	No, Open label	Yes	Lir 1.2/Lir 1.8/Sita: 75.1/86.4/88.6	Yes	Yes	Yes

Marre 2009 (LEAD-1)[39]	Unclear	Unclear	Yes, Double blind	Yes	Lir 1.2/Lir 1.8/Rosi/P: 86.0/91.0/83.6/72.8	Yes	Yes	Yes

Nauck 2009 (LEAD-2)[36]	Yes	Yes	Yes, Double blind	Yes	Lir 1.2/Lir 1.8/Glim/P: 82.1/78.9/86.8/61.2	Yes	Yes	Yes

Zinman 2009 (LEAD-4)[44]	Yes	Yes	Yes, Double blind	Yes	Lir 1.2/Lir 1.8/P: 86.0/74.7/68.4	Yes	Yes	Yes

Russell-Jones 2009 (LEAD-5)[35]	Yes	Yes	Unclear	Yes	Lir 1.8/Glar/P: 90.0/94.4/84.2	Yes	Yes	Yes

Buse 2009 (LEAD-6)[40]	Unclear	Yes	No, Open label	Yes	Lir 1.8/Exe: 86.7/81.0	Yes	Yes	Yes

Nauck 2009[24]	Yes	Yes	Yes, Double blind	Yes	Tas 10 mg QW/Tas 20 mg QW/Tas 20 mg every two weeks/P: 91.8/88.0/93.9/95.9	Yes	Yes	Yes

Ratner 2010[25]	Yes	Unclear	Yes, Double blind	Yes	Tas 20 mg QW/Tas 30 mg QW/Tas 40 mg QW: 90.6/81.8/81.3/96.9	Yes	Yes	No

Albi: Albiglutide; Exe: Exenatide; Exe lar: long acting exenatide; Lir: Liraglutide; Tas: Taspoglutide; BIAsp: Biphasic insulin aspart; Glar: Insulin glargine; Ins: Insulin; Glib: Glibenclamide; Glim: Glimepiride; Pio: Pioglitazone; Rosi: Rosiglitazone; Sita: Sitagliptin; P: Placebo; BID: Twice daily; QD: Once daily; QW: Once weekly; HEELA: Helping Evaluate Exenatide in patients with diabetes compared with Long-Acting insulin; LEAD: Liraglutide Effect and Action in Diabetes

## Results

### HbA1c

**Figure 2:**
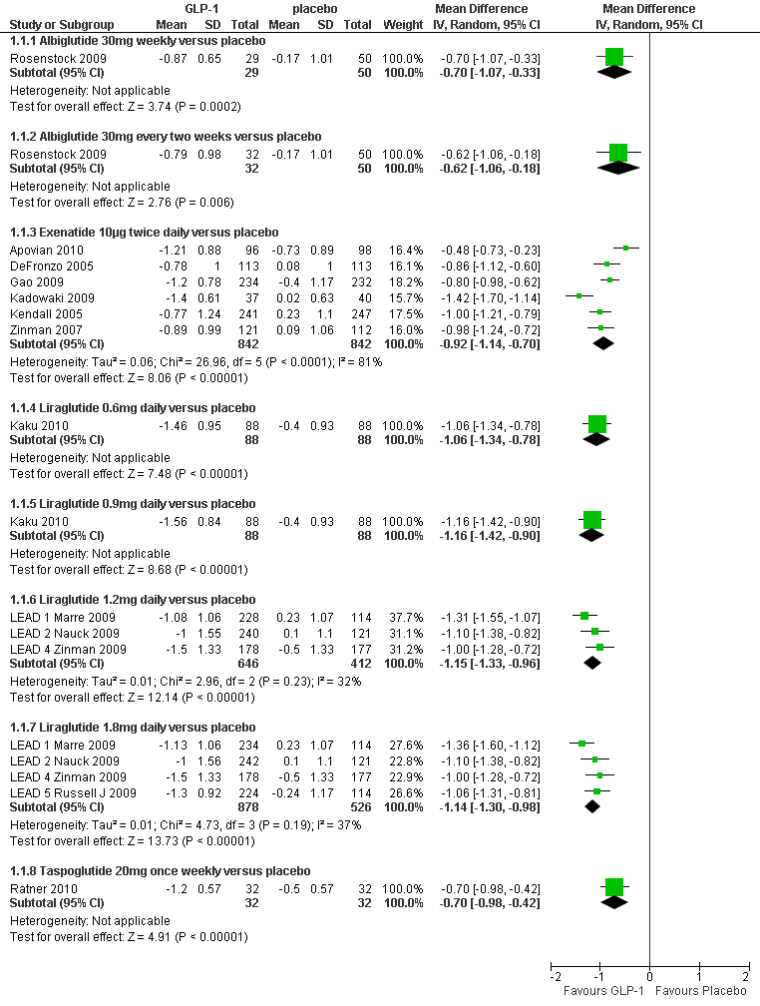
**Change in HbA1c (%): GLP-1agonists versus placebo**.

Figure 2 shows the meta-analysis of the percentage change in HbA1c for the GLP-1 agonist trials in comparison with placebo. It can be seen that all GLP-1 agonists significantly reduced HbA1c, with reductions ranging from 0.62% to 1.16%. Two studies[24,26] could not be included in the meta-analysis due to insufficient details reported. Gill 2010[26] compared exenatide 10 µg twice daily versus placebo. The mean difference between groups was not significant (0.3% SD 1.06). However, this trial was a small, short term trial powered to detect differences in mean 24-hr heart rate (not HbA1c). Nauck 2009[24] compared taspoglutide at doses of 5 mg, 10 mg and 20 mg once weekly, and 10 mg and 20 mg once every two weeks, and found all doses changed significantly compared to placebo. This study could not be included in the meta-analysis because of lack of data on standard deviations (SDs) or standard errors (SEs).

**Figure 3:**
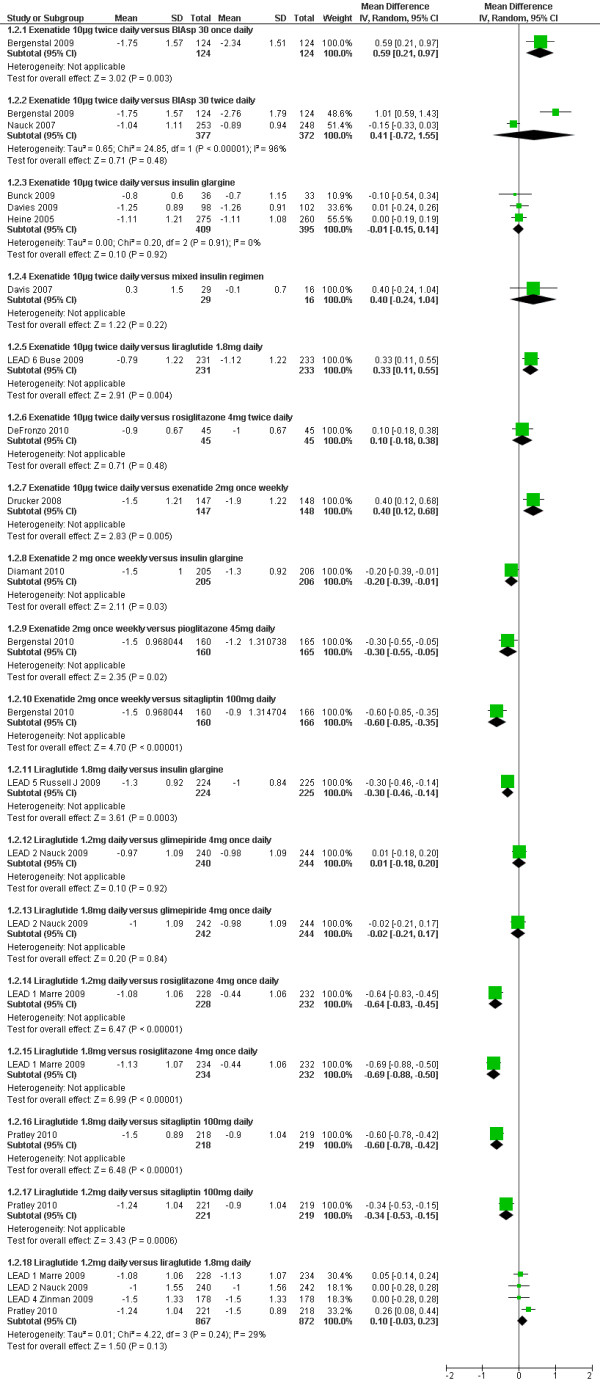
**Change in HbA1c (%): GLP-1 agonists versus active comparators**.

The results of forest plots of GLP-1 agonists against active comparators are shown in Figure 3. Derosa 2010[27] could not be included due to insufficient details (SDs) reported.

#### GLP-1 agonists versus insulin

Six RCTs[28-33] compared exenatide (10µg twice daily) with insulin (BIAsp, glargine or mixed insulin regimens), one[34] compared long acting exenatide (2 mg once weekly) with glargine and one[35] compared liraglutide with glargine. Two studies[28,33] compared BIAsp 30 twice daily with exenatide twice daily. Nauck 2007[33] found a non significant difference in favour of exenatide, but Bergenstal 2009[28] reported a significantly greater reduction in HbA1c with BIAsp 30 twice daily (1.0%, p < 0.00001) and once daily (0.59%, p = 0.003). Bergenstal 2009 was sponsored by Novo Nordisk, the manufacturer of BIAsp30, and the study by Nauck 2007 was sponsored by Lilly, the manufacturer of exenatide. The explanation for the highly significant heterogeneity of I^2 ^= 96% between Bergenstal 2009 and Nauck 2007 may reflect differences in baseline HbA1c (10.2% vs. 8.6%) and BMI (33.8 vs. 30.4) respectively.

None of the three studies comparing exenatide with insulin glargine (Bunck 2009, Davies 2009 and Heine 2005)[29,30,32] found a significant difference, and nor did Davis 2007[31], which compared patients remaining on their existing insulin regimen with patients switching to exenatide. Diamant 2010[34] found a slightly greater reduction in HbA1c with exenatide 2 mg once weekly than with insulin glargine (-1.5 vs. -1.3%; p = 0.03). Liraglutide 1.8 mg daily caused a slightly greater reduction in HbA1c than did insulin glargine (-1.3 vs. -1.0%; p = 0.0003) in the Russell-Jones 2009[35] (LEAD-5) trial, but the dose of glargine used may have been sub-optimal.

#### GLP-1 agonists versus sulphonylureas

Derosa 2010[27] found similar reductions in HbA1c level for exenatide compared with glibenclamide 2.5 mg three times daily (-1.50 vs. -1.80%) and Nauck 2009[36] (LEAD-2) found no significant difference between either liraglutide 1.2 or 1.8 mg daily and glimepiride 4 mg daily; HbA1c was reduced by 1% in all treatment groups.

#### GLP-1 agonists versus pioglitazone

Bergenstal 2010[37] found a slightly greater reduction in HbA1c level with exenatide 2 mg once weekly than with pioglitazone 45 mg once daily (-1.5 vs. -1.2%; p = 0.02).

#### GLP-1 agonists versus sitagliptin

Bergenstal 2010[37] found a significantly greater reduction in HbA1c with exenatide 2 mg once weekly than sitagliptin 100 mg once daily (-1.5 vs. -0.9%; p < 0.00001). Pratley 2010[38] found that liraglutide 1.2 mg daily led to significantly greater reduction in HbA1c level than with sitagliptin 100 mg once daily (-1.24 vs. -0.9%; p < 0.00001).

#### GLP-1 agonists versus rosiglitazone

DeFronzo 2010[22] found similar reductions in HbA1c level for exenatide compared with rosiglitazone 4 mg twice daily (-0.9 vs. -1.0%). By contrast, the Marre 2009[39] (LEAD-1) trial found that the reduction in HbA1c level was significantly greater with liraglutide 1.2 mg and 1.8 mg daily compared with rosiglitazone 4 mg daily (-1.08 and -1.13 vs. - 0.44%; p < 0.0001).

#### GLP-1 agonists versus GLP-1 agonists

In the Buse 2009[40] (LEAD-6) trial, liraglutide 1.8 mg daily led to significantly greater reduction in HbA1c than exenatide 10 μg twice daily (-1.12% vs. - 0.79%; p = 0.004). Drucker 2008[41] found that long acting exenatide (2 mg once weekly) was found to cause a greater reduction in HbA1c than exenatide 10 μg twice daily (-1.9% vs. -1.5%, mean difference 0.40 (95% CI: 0.12, 0.68), p = 0.005).

The meta-analysis of the trials involving arms with both 1.2 mg and 1.8 mg daily doses of liraglutide showed a non-significant difference of -0.10% (95% CI -0.03 to 0.23) in favour of the 1.8 mg dose.

### Percentage of patients achieving HbAlc ≤7%

**Figure 4:**
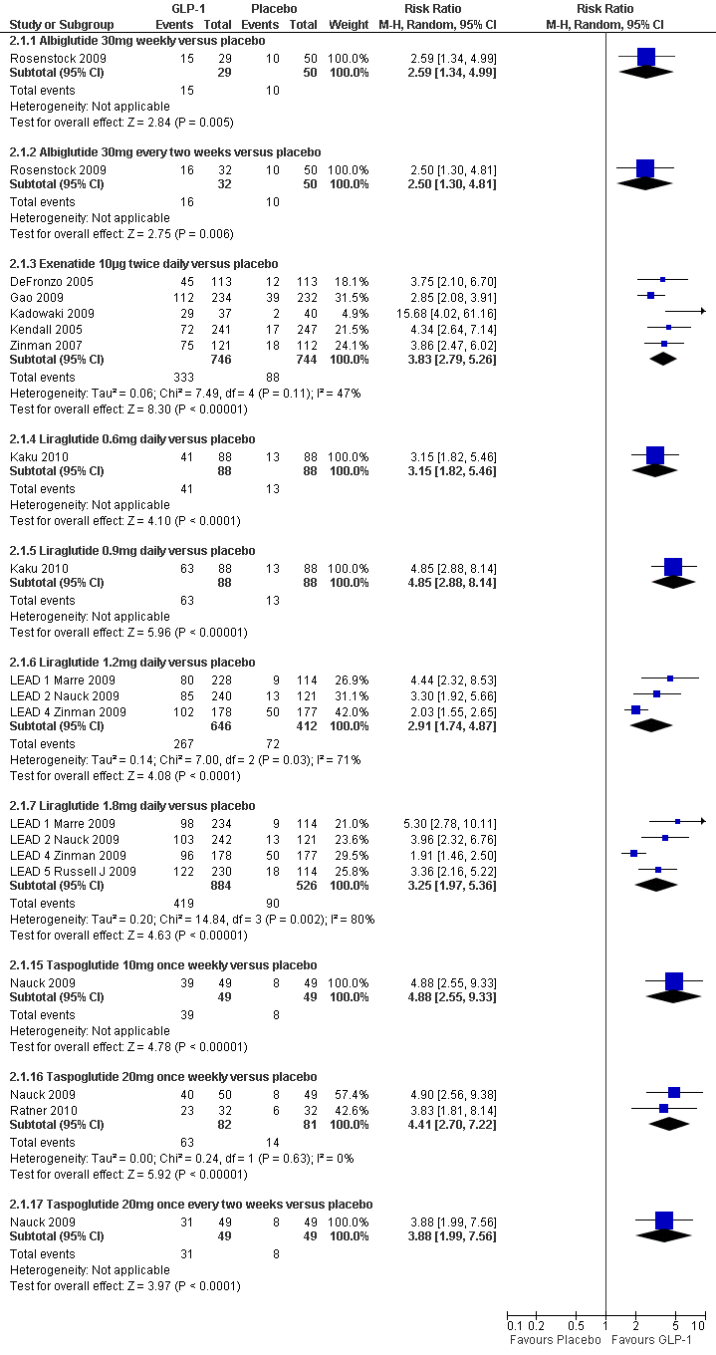
**Percentage of patients achieving HbA1c ≤7%:GLP-1 agonists versus placebo**.

**Figure 5:**
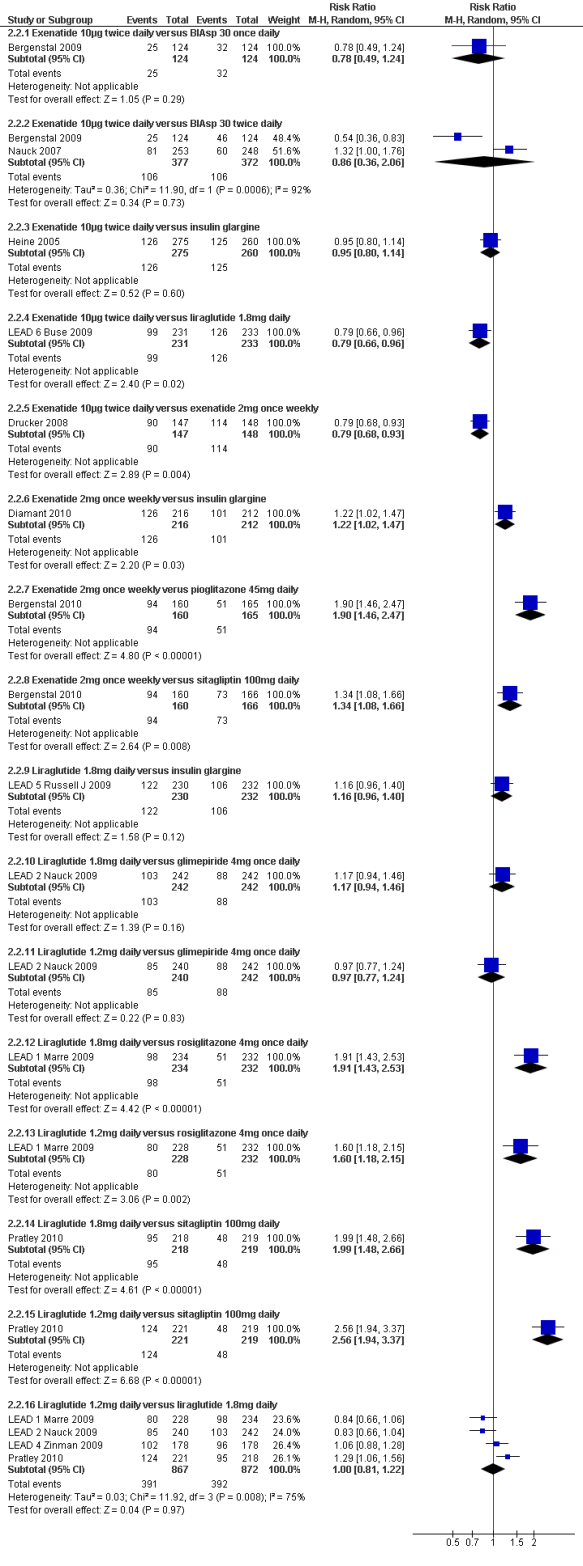
**Percentage of patients achieving HbA1c ≤7% GLP-1 agonists versus active comparators**.

As expected, the proportion of participants achieving an HbA1c level of 7% or less was significantly greater with all GLP-1 agonists compared to placebo (shown in Figure 4). As with the overall HbA1c reduction, there are suggestions that effect sizes vary amongst GLP-1 agonists. Figure 5 shows the risk ratios of the proportion of participants achieving a target HbAlc level of ≤7% with GLP-1 agonists compared to an active comparator. Five studies (Gill 2010, Bunck 2009, Davis 2007, Derosa 2010, and DeFronzo 2010)[22,26,27,29,31] could not be included in these meta-analyses due to the relevant data not being reported. The data for Bergenstal 2010[37] was estimated from the graph.

#### GLP-1 agonists versus insulin

As with the results for changes in HbA1c, there was highly significant heterogeneity when combining the two trials[28,33] comparing BIAsp 30 twice daily and exenatide. Bergenstal 2009 reported significantly fewer patients achieving a target HbA1c level of ≤ 7% with exenatide compared to BIAsp 30 twice daily group (20% vs. 37%, p = 0.004), while Nauck 2007 reported a higher number of patients achieving this target with exenatide (32% vs. 24%; p = 0.05).

Heine 2005[32] found no significant difference between exenatide and glargine (46% vs. 48%), and Russell Jones 2009[35] (LEAD-5) found no significant difference between liraglutide 1.8 mg daily and glargine (53% versus 46%). Diamant 2010[34] found that exenatide 2 mg once weekly led to a slightly greater proportion of patients achieving this target than with insulin glargine (60% vs. 48%; p = 0.03)

#### GLP-1 agonists versus sulphonylureas

There was no significant difference between liraglutide 1.2 mg or 1.8 mg daily and glimepiride (35% or 42% versus 36%)[36].

#### GLP-1 agonists versus pioglitazone

Bergenstal 2010[37] found that exenatide 2 mg once weekly led to significantly higher number of patients achieving HbA1c level of ≤7% than with pioglitazone 45 mg daily (59% vs. 31%).

#### GLP-1 agonists versus sitagliptin

Bergenstal 2010[37] found that significantly greater number of patients achieved a target HbA1c level of ≤7% with exenatide 2 mg once weekly than with sitagliptin 100 mg daily (59% vs. 44%; p = 0.008). Russell Jones 2009[35] (LEAD-5) found that the proportion of participants achieving this target was significantly higher with liraglutide 1.2 mg daily and sitagliptin (56% vs. 22%).

#### GLP-1 agonists versus rosiglitazone

The proportion of participants achieving HbA1c level of ≤7% was significantly higher with liraglutide 1.2 mg daily compared to rosiglitazone 4 mg daily (35% vs. 22%)[39].

#### GLP-1 agonists versus GLP-1 agonists

Drucker 2008[41] reported that exenatide 2 mg weekly was superior to the 10 µg twice daily dose (77% vs. 61%; p = 0.004), and liraglutide 1.8 mg daily was significantly better than exenatide 10 μg twice daily (54% vs. 43%; p = 0.02). There was no significant difference between the liraglutide 1.2 mg and 1.8 mg dose.

### Body weight

**Figure 6:**
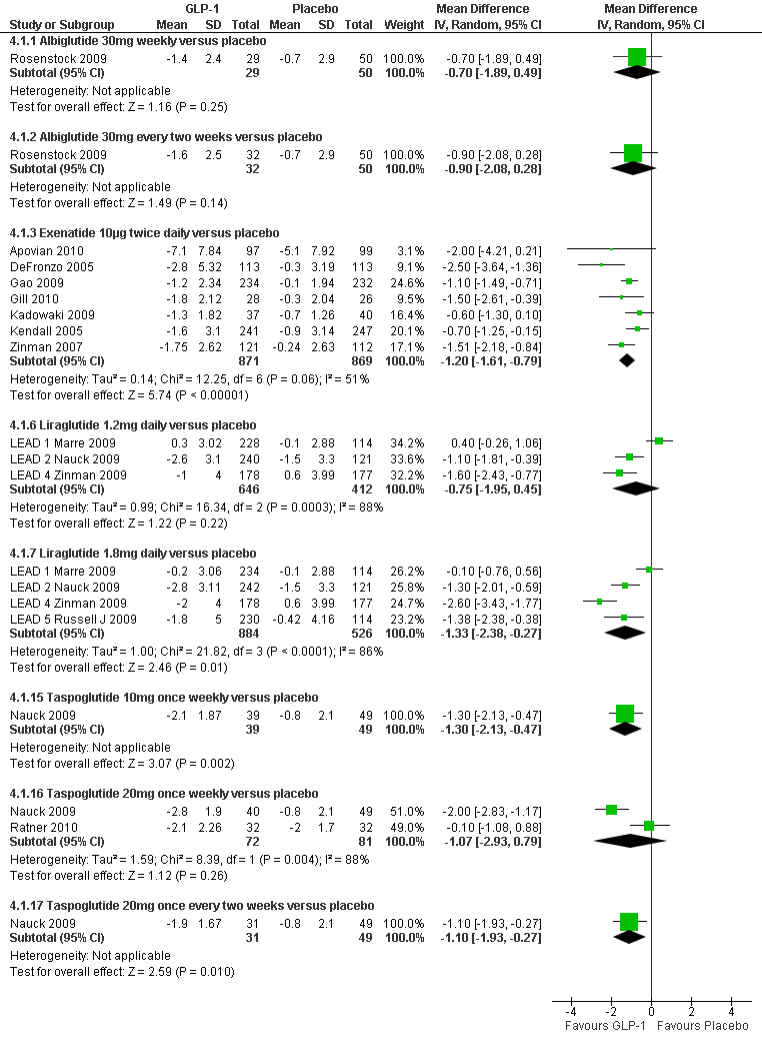
**Weight changes (kg): GLP-1 agonists versus placebo**.

As shown in Figure 6, mean weight losses with GLP-1 agonists ranged from 0.7 to 1.3 kg. Several trials reported that weight loss occurred in patients not experiencing nausea[21,32,35,36,41,42]. All GLP-1 agonists caused greater weight loss compared with placebo, apart from the Marre 2009[39] (LEAD-1) trial, where the liraglutide 1.2 mg arm gained 0.3 kg, and the placebo arm lost 0.1 kg; the differences were not statistically significant.

**Figure 7:**
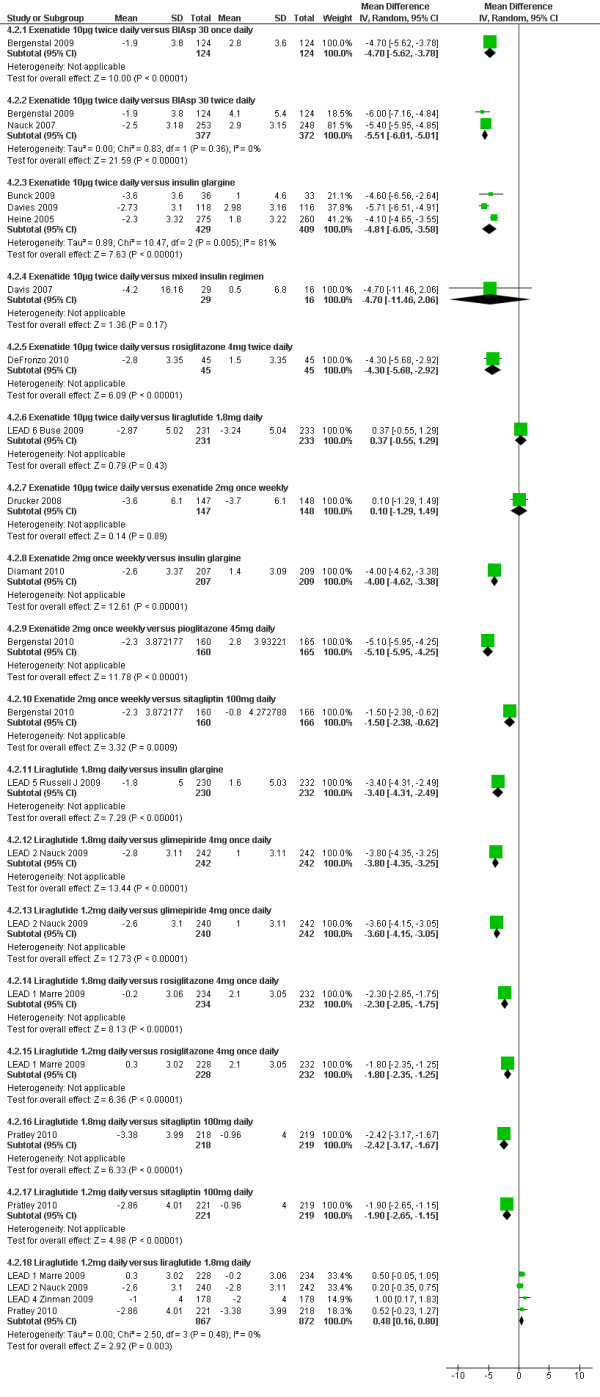
**Weight changes (kg): GLP-1 agonists versus active comparators**.

As shown in Figure 7, greater relative weight loss occurred in trials of GLP-1 agonists against active comparators than with trials against placebo, as all of the non GLP-1 active comparators except sitagliptin tended to cause weight gain.

#### GLP-1 agonists versus insulin

Patients in the exenatide versus insulin trials lost between 1.9 and 4.2 kg of weight, whereas patients in the insulin groups gained between 0.5 and 4.1 kg of weight. The meta-analysis showed a significant difference in favour of exenatide for BIAsp30 insulin and insulin glargine, and a non-significant loss against a mixture of insulin regimens. Diamant 2010[34] showed a significantly greater reduction in weight with exenatide 2 mg once weekly and an increase with insulin glargine (-2.6 vs. +1.4 kg; p < 0.00001). In Russell-Jones 2009[35] (LEAD-5), liraglutide was found to cause significant weight reduction (-1.8 kg) compared to an increase (+1.6 kg) with insulin glargine.

#### GLP-1 agonists versus sulphonylureas

Exenatide was also found to cause weight loss compared to gains with glibenclamide (-8 kg vs. +4.30 kg; p < 0.001) in Derosa 2010[27]. In Nauck 2009[36] (LEAD-2), liraglutide 1.2 mg and 1.8 mg showed significant weight loss compared to glimepiride (-2.6 to -2.8 kg vs. +1 kg; p < 00001).

#### GLP-1 agonists versus pioglitazone

Exenatide 2 mg once weekly led to weight loss whereas pioglitazone 45 mg daily caused weight gain (-2.3 vs. +2.8 kg; p < 0.00001)[37].

#### GLP-1 agonists versus sitagliptin

In Bergenstal 2010[37], exenatide led to a significantly greater weight loss with exenatide 2 mg once weekly than with sitagliptin 100 mg daily (-2.3 vs. -0.8 kg; p = 0.0009).

#### GLP-1 agonists versus rosiglitazone

In DeFronzo 2010[22], exenatide was found to cause weight loss, compared to a gain with rosiglitazone 4 mg twice daily (-2.8 kg vs. +1.5 kg, p < 0.001). Similarly in Marre 2009[39], liraglutide 1.2 mg and 1.8 mg led to significant weight loss compared to rosiglitazone 4 mg once daily (-0.2 and +0.3 kg vs. +2.1 kg, p < 0.0001).

#### GLP-1 agonists versus GLP-1 agonists

Drucker 2008[41] compared twice daily exenatide versus once weekly exenatide, and found no difference in weight loss or the proportions losing weight (76% with exenatide once weekly versus 79% with exenatide twice daily). Weight decreased in participants who reported no episodes of nausea throughout the study (70%). In Buse 2009[40] the amount of weight loss was similar with exenatide and liraglutide (-2.87 kg vs. -3.24 kg), as was the proportion of participants who lost weight (78% vs. 76%). The 1.8 mg dose of liraglutide gave slightly greater weight loss than the 1.2 mg dose, with overall mean difference of 0.48 kg (95% CI 0.16 to 0.80).

### Fasting and postprandial plasma glucose (FPG and PPG)

As shown in additional file 1 all GLP-1 agonists significantly reduced FPG compared with placebo, partly because most of the placebo groups showed a small increase in FPG.

The overall difference between trials comparing exenatide 10 μg twice daily and insulin glargine in FPG was 1.37 mmol/l [95% CI 1.01 to 1.73] (with a heterogeneity of I^2 ^= 0%) in favour of glargine[29,30,32]. However, the trial of liraglutide 1.8 mg daily versus glargine showed a non-significant difference of 0.24 mmol/l (95% CI -0.34 to 0.82) in favour of liraglutide[35].

Liraglutide reduced FPG significantly more than exenatide 10 μg twice daily by 1.01 mmol/l (95% CI 0.46 to 1.56)[40]. Long acting exenatide (2 mg weekly) caused a significantly greater reduction of 0.90 mmol/l (95% CI 0.35 to 1.45) in FPG compared to exenatide 10 μg twice daily. Bergenstal 2010[37] found a significant difference in favour of exenatide 2 mg once weekly compared with sitagliptin 100 mg daily (-0.90 mmol/l, 95% CI -1.50, -0.30; p = 0.0038) but a non-significant difference against pioglitazone 45 mg daily (-0.30 mmol/l, 95% CI -0.90 to 0.30; p = 0.33).

In the studies that reported on PPG, the values were derived differently in each study. PPG was lower in all GLP-1 groups compared to placebo, and there was significantly less variability in results from self monitoring blood glucose (SMBG).

In one head to head trial[40], exenatide led to a greater reduction in PPG increment than liraglutide after breakfast and dinner (treatment differences were: breakfast 1.33 mmol/L, 95% CI: 0.80 to 1.86, p < 0.0001; dinner 1.01 mmol/L, 95% CI: 0.44 to 1.57, p = 0.0005) however the difference was not significant after lunch, which fits with the time of administration[40]. Exenatide 10 µg twice daily was superior to the once weekly 2 mg regimen in controlling PPG. Exenatide 2 mg once weekly showed significantly greater reduction in PPG than did sitagliptin (p < 0.05) but not compared to pioglitazone[37]. Exenatide 2 mg once weekly led to greater reduction in post prandial glucose excursions after morning (p = 0.001) and evening (p = 0.033) meals than insulin glargine[34].

### Blood pressure

In all but one trial[18], GLP-1 agonists led to reductions in SBP of a few mmHg. In most studies, the differences in reduction in DBP with GLP-1 s were not significant (see additional file 1).

### Lipid profile

No significant changes were seen in the lipid profiles of the participants treated with either albiglutide or placebo. Five exenatide trials[20,22,30,41,43] reported results for lipid profiles. No significant reductions in lipid parameters were seen in comparison with either placebo or insulin. Exenatide 10 μg twice daily however showed significant reduction (-0.13 vs. +0.44; p < 0.001) in total cholesterol (TC) and LDL (-0.05 vs. +0.33; p = 0.008) in comparison with rosiglitazone[22]. Once-weekly long acting exenatide led to greater reduction in TC (-0.31 vs. -0.10) and LDL (-0.13 vs. +0.03) than twice daily exenatide[41]. Bergenstal 2010[37] found that all drugs improved HDL levels, but only pioglitazone reduced triglycerides significantly. There was a reduction in the total cholesterol level and LDL with exenatide 2 mg once weekly, but an increase with pioglitazone and sitagliptin. The differences were not significant. Diamant 2010[34] found that exenatide 2 mg once weekly led to slightly greater reduction in TC and LDL than with glargine (-0.12 vs. -0.04 mmol/l TC; -0.05 vs. +0.04 mmol/l LDL). There was no difference in triglyceride levels.

Only three liraglutide trials[38,40,44] reported lipid profiles in detail and one[18] reported briefly. In LEAD-4[44], there was significantly more reduction in triglycerides, TC and LDL cholesterol with 1.2 mg daily liraglutide (but, oddly, not with 1.8 mg liraglutide) than with placebo. There were no significant differences in the lipid profiles between liraglutide 1.8 mg and sitagliptin, apart from a modest decrease -0.16 mmol/l (95% CI -0.30 to -0.01) in total cholesterol, in favour of liraglutide[38]. Kaku 2010[18] compared liraglutide 0.6 mg and 0.9 mg and reported no significant changes in lipid profiles. In the LEAD 6 trial[40], liraglutide 1.8 mg led to a significantly greater reduction in triglycerides (TG) -0·18 (95% CI -0·37 to 0·00) than exenatide 10 μg twice daily. There were no significant differences in any other lipid parameters. Taspoglutide caused a dose-related decline in TG levels and a small decrease in TC levels. None of the other lipid parameters showed any consistent changes.

### Beta cell function

All GLP-1 agonists led to significant improvements in beta-cell function, but these did not persist once users stopped the drug[29]. The improvement with GLP-1 agonists was better than with glargine and rosiglitazone, but no different from glimepiride[36]. There was no difference between liraglutide and exenatide[40].

### Treatment satisfaction/Quality of life

Only two trials[34,37] reported quality of life using weight related quality of life or Impact of Weight on Quality of Life (IWQOL) and EuroQoL questionnaires. Also both studies used the Diabetes Treatment Satisfaction Questionnaire (DTSQ) to assess the overall treatment satisfaction. Overall treatment satisfaction was also reported by one exenatide[41] and two liraglutide trials [38,40]. All three studies used the DTSQ. In the two liraglutide trials, only subgroups of patients completed the questionnaires.

Bergenstal 2010[37] found that all the five parameters of weight-related QOL and IWQOL total score significantly improved with exenatide 2 mg once weekly (IWQOL total score 5.15, 95% CI 3.11 to 7.19) and sitagliptin (4.56, 95% CI 2.56 to 6.57) but not with pioglitazone (1.20, 95% CI -0.87 to 3.28). The improvement in IWQOL total score with exenatide was consistent with differences in body weight changes. Overall treatment satisfaction was also higher with exenatide 2 mg once weekly than with sitagliptin (3.96 vs. 2.35; difference 1.61, 95% CI 0.07 to 3.16; p = 0.0406). Diamant 2010[34] reported significant improvements for one of the IWQOL-Lite domains and one EQ-5D dimension with exenatide 2 mg once weekly compared with insulin glargine, but no data were given.

There was no significant difference in treatment satisfaction between the exenatide once a week and the exenatide twice daily groups [changes observed using DTSQ; exenatide once weekly 0.84 vs. exenatide twice daily 0.64; p = 0.09][41]. Overall satisfaction was higher with liraglutide 1.8 mg than with exenatide 10 μg twice daily, but while the difference was statistically significant (liraglutide 1.8 mg 15.18 SE 0.58 vs. exenatide 13.30 SE 0.58; estimated treatment difference 1.89 (95% CI 0.85 to 2.92); p = 0.0004) the difference of less than two points was small on the 36-point scale[40]. Similarly, there was little difference between liraglutide and sitagliptin (DTSQ; liraglutide 1.2 mg vs. sitagliptin p = NS; liraglutide 1.8 mg vs. sitagliptin change from baseline 1.39 on the 36 point scale (95% CI 0.13 to 2.64), p = 0.03), implying that patients did not find that having to inject liraglutide was a problem[38].

### Hypoglycaemia

The definition of hypoglycaemia was given in most studies. Minor hypoglycaemia was defined as an episode that could be self-treated, while those needing third party assistance or medical interventions were categorised as major. Results showing the incidence of hypoglycaemia are shown in additional file 2.

The incidence of minor hypoglycaemia was significantly greater in trials where patients were taking exenatide in combination with sulphonylureas[20,21,43,45] compared to those not taking sulphonylureas. Minor hypoglycaemia was higher with liraglutide than rosiglitazone in Marre 2009[39] (LEAD -1) (where treatment included sulphonylureas in both arms), and in Nauck, 2009[36] (LEAD-2) it was higher with glimepiride than liraglutide (17% vs. 3%).

The incidence of major hypoglycaemia was low with GLP-1 s. Davis 2009[30] reported three episodes in one patient taking exenatide in combination with a sulphonylurea, and none in the insulin group. Bunck 2009[29] reported more overall hypoglycaemia with glargine (24.2% vs. 8.3%) than exenatide, but there was no severe hypoglycaemia in either group. In the Davies 2009[30] and Heine 2005[32] trials, the rates of overall and severe hypoglycaemia were similar, but in the latter trial, the rate of nocturnal hypoglycaemia was higher with glargine than with exenatide. Diamant 2010[34] reported a greater number of patients with minor hypoglycaemia in the insulin glargine group than the exenatide 2 mg once weekly group (26 vs. 8%). Two patients (one taking metformin only and the other taking metformin plus sulphonylurea) in the glargine group and one in the exenatide 2 mg once weekly group (taking metformin only) had major hypoglycaemia.

In the Marre 2009[39] (LEAD 1) trial, one patient taking liraglutide 1.8 mg and glimepiride had major hypoglycaemia, and this was considered to be related to glimepiride. In Russell-Jones, 2009[35] (LEAD 5), five patients had major hypoglycaemic events in the liraglutide group, and in Buse 2009[40] (LEAD 6) patients in the exenatide group reported two episodes of major hypoglycaemia, whereas none were found in the liraglutide group. Pratley 2010[38] reported that one patient on 1·2 mg liraglutide had a major hypoglycaemic episode, but none on the 1.8 mg dose or on sitagliptin. The other liraglutide trials reported no severe hypoglycaemia in any group[18,36,44].

There were no significant differences in the incidence of hypoglycaemia in trials comparing albiglutide or taspoglutide and placebo.

### Adverse events

Details of adverse events are shown in additional file 2. The most commonly reported adverse events were nausea, vomiting and diarrhoea, and withdrawals from the trials were mostly because of these events. In head to head trials comparing one GLP-1 agonist against another (exenatide vs. liraglutide and exenatide twice daily vs. exenatide once weekly) there were no significant differences in initial frequency[40,41], but nausea was less persistent with liraglutide compared to exenatide[40]. Nausea and vomiting were less with once weekly exenatide compared with twice daily exenatide[41]. Most studies stated that gastrointestinal effects were worst at the beginning and tended to be reduced over the course of the study. There were reports of mild to moderate injection site reactions, which did not lead to any discontinuation.

Only one case of pancreatitis was reported with exenatide[29] however it resolved after stopping the medication. Similarly, one case of acute pancreatitis was seen with 1.2 mg liraglutide[36] and the patient withdrew from the study. Another patient taking liraglutide 1.8 mg developed chronic pancreatitis; however the investigators considered that it was unrelated to the drug[40].

## Discussion

This systematic review and meta-analysis included twenty eight randomised controlled trials and compared four different GLP-1 agonists against placebo and a number of active comparators. The comparisons were: albiglutide and taspoglutide against placebo; exenatide against placebo, insulin, glibenclamide, rosiglitazone; exenatide twice daily against exenatide once weekly; exenatide once weekly against sitagliptin and pioglitazone; and liraglutide against placebo, exenatide, glimepiride, rosiglitazone, sitagliptin and insulin glargine. All but one of the trials were sponsored by the manufacturers, and where details are given, the manufacturers were involved in, or carried out, data analysis.

### Summary of principal findings

The results showed that GLP-1 agonists are effective in improving glycaemic control and promoting weight loss, with a low risk of hypoglycaemia, and can be an alternative to immediate insulin in patients failing on combined oral glucose lowering agents.

#### HbA1c

Results varied against active comparators. Liraglutide 1.8 mg daily was superior to glargine, rosiglitazone 4 mg daily, sitagliptin 100 mg daily and exenatide 10 μg twice daily. Exenatide 10 μg twice daily was equivalent to both insulin and rosiglitazone 4 mg twice daily, taking differences in HbA1c of less than 0.5% as being not clinically significant. Long acting exenatide (2 mg weekly) was superior to exenatide 10 μg twice daily, glargine, sitagliptin and pioglitazone 45 mg daily

#### Weight loss

Exenatide and liraglutide caused greater weight loss than all active comparators, most of which led to weight gain. Weight loss was independent of nausea. A study that followed trial patients for longer has shown that temporal patterns of weight loss can vary amongst patients.[46]

#### Hypoglycaemia and adverse events

The incidence of major hypoglycaemia was very low (in absolute terms) in patients taking GLP-1 agonists, and the incidence of minor hypoglycaemia was low (under 10%) compared to most other glucose lowering agents except metformin. Hypoglycaemia was seen most often when GLP-1 analogues were used in combination with sulphonylureas, in which cases it was probably due to those rather than the GLP1 agonist. The most commonly reported adverse events with GLP-1 agonists were gastrointestinal, and included nausea, vomiting and diarrhoea. These adverse events were worst at the beginning and reduced over the course of therapy. Most patients did not get nausea while taking a GLP-1 agonist.

### Strengths and Limitations

This review carried out a systematic search to identify all the relevant papers on the effectiveness of GLP-1 agonists in patients with type 2 diabetes who are inadequately controlled with oral hypoglycaemic agents. All the included studies are randomised controlled trials comparing GLP-1 agonists against a placebo, an active comparator or other GLP-1 agonists. We have only included trials that have used a clinically relevant dose of a GLP-1 agonist. We excluded trials of GLP-1 analogues against placebo in patients on no other glucose lowering drug, because in practice, older cheaper drugs with long safety records, such as metformin, should be used first. Also, we have only included clinically relevant comparators, and excluded patients who were naïve to therapy or who were treated with a GLP-1 agonist as monotherapy. Wherever possible, the data were meta-analysed. We have separately analysed GLP-1 agonists versus placebo, and GLP-1 agonists versus active comparators.

This review includes searches up to July 2010, and hence includes the newest GLP-1 agonists, such as taspoglutide, albiglutide and long acting exenatide.

#### Limitations to our review

We did not include data from observational studies in the main results. Some of these studies have suggested that results in routine care are not as good as in the RCTs. For example, Loh and Clement found no significant improvement in HbA1c at two years, and that most of the weight lost was regained [47]. However a subgroup did well on both parameters. Observational studies may be useful for identifying those patients most likely to do well on the GLP-1 agonists. However, most studies from routine care are available only as conference abstracts.

We included only those doses which were licensed, or in the case of unlicensed drugs, we included doses which seemed from the trial results, or from the authors' comments, to be the ones likely to be used in clinical practice.

In the meta-analyses, we grouped trials comparing GLP-1 agonists versus comparators, irrespective of the co-medications used (as long as the co-medications were the same across arms). This assumed that the effect size of GLP-1 agonists against comparators does not depend on whether the GLP-1 agonists are used as a second drug or a third drug. We investigated the effect of co-medications in the liraglutide trials, some of which used liraglutide in dual and some in triple therapy. The proportion of patients who had received previous dual therapy ranged from zero to 84%. However, there was no systematic difference in reductions in HbA1c between the dual and triple therapy trials. There were also, as mentioned above, uncertainties or lack of data on which medications patients had had before entry.

Most limitations reflected gaps in the evidence, including the following.

#### Head to head comparisons of GLP-1 agonists

There is currently only one trial comparing one GLP-1 agonist against another (liraglutide versus exenatide), and no firm conclusions can be made on the relative effectiveness of different GLP-1 agonists.

Exenatide 2 mg weekly was superior to exenatide 10 µg twice daily with respect to HbA1c. In the head-to-head comparison of exenatide 10 μg twice daily and liraglutide, liraglutide was superior to exenatide. This trial was sponsored by the manufacturer of liraglutide, but the findings appear plausible and in keeping with the pharmacodynamics of the two drugs, with liraglutide having a more prolonged action with less of a "peak and trough" effect. The trial of weekly exenatide versus twice daily exenatide showed that the weekly version reduced HbA1c by 0.4% more than the twice daily one, so an indirect comparison would suggest that weekly exenatide might be more potent than daily liraglutide. Hence, the future of the GLP-1 agonists would appear to be as a medication given weekly, or even every two weeks.

The only advantage of the twice daily formulation of exenatide compared with the weekly one, was that it gave better control of PPG. There has been concern that post-prandial excursions might be an independent (of overall control) risk factor for complications, but this is unproven[48]. Diamant 2010[34] showed better control of PPG with exenatide once weekly than with glargine.

Our meta-analysis showed that there was little advantage of the 1.8 mg dose over the 1.2 mg dose of liraglutide, with no difference in HbA1c, but slightly greater weight loss.

At present, any comparison amongst GLP-1 agonists would have to be done by indirect comparison, which could only be done using studies where co-medications were the same, and where there was a common comparator. Indirect comparisons are not as good as head to head trials, but are sometimes the best that can be done if such trials are not available, and are hence suggested by the National Institute for Health and Clinical Excellence's (NICE) Guide to the methods of technology appraisal June 2008[49].

#### Lack of trials against, or in combination with, insulin

Only four exenatide trials and one liraglutide trial made comparisons against insulin glargine, the most commonly used basal insulin in the UK. There have been no trials against NPH insulin, which is more cost-effective in type 2 diabetes than the long-acting analogues[50].

There were no trials of combinations of basal insulin and GLP-1 agonists. Given their different actions, such combinations seem logical, but are currently unlicensed. Observational studies suggest their safety and efficacy [51].

#### Lack of trials against pioglitazone

There is only one trial of a GLP-1 agonists against pioglitazone, which because of its better cardiovascular risk profile than rosiglitazone, is now preferable[50].

#### Length of trials

Only two studies lasted 52 weeks - so there is insufficient evidence regarding long-term outcomes. Two studies lasted only 8 weeks. The studies included in this review were not long enough to entirely remove concerns about pancreatitis and renal failure[52-54] with exenatide or pancreatitis and thyroid carcinoma[55] with liraglutide. There have been reports of pancreatitis with exenatide and liraglutide, but it is difficult to prove if the drugs are responsible because the incidence of pancreatitis is increased in type 2 diabetes[56].

Type 2 diabetes is usually a progressive disease, and trials to date have not been long enough to tell us for how long the GLP-1 agonists would be effective. Short duration of the trials do not provide data on cardiovascular outcomes[57].

#### Losses to follow-up

Many of our inclusions had one or more arms with losses to follow up of > 20%. Ideally, trials with an attrition rate of between 5 and 20% are acceptable, but greater proportions may pose a threat to validity[58,59].

#### Doses of comparators used in trials

In the LEAD-1 trial of liraglutide against rosiglitazone, the 4 mg daily dose was used. The manufacturer, Novo Nordisk, explained that this was because the trial was done in a number of countries, in some of which the 8 mg dose is not licensed. It is possible that using the larger dose might not have made much difference. There is one trial comparing the two doses, by Fonseca et al, in which the HbA1c gain from the larger dose was only 0.22%[60].

In the trial by Pratley et al, 22% of patients achieved an HbAlc level ≤7% on sitagliptin[38]. In LEAD 1, 22% reached the same target on 4 mg daily rosiglitazone[39]. Hence, it appears worth trying both of those agents before a GLP-1 agonist, because almost a quarter of patients will achieve good control, at least in the short term.

### Previous reviews

There have been several good quality reviews, including those by Monami and colleagues [11], Barnett [10], Amori and colleagues [9], and Norris and colleagues [12], and a health technology assessment (HTA) report[50]. However, some include all comparators in all trials (i.e. not all are relevant to clinical practice), and none include trials published up to July 2010. Also, some reviews did not include all the GLP-1 agonists.

### Beta-cell function

As beta- cell capacity declines, some of the GLP-1 agonist effects would be lost, but others (such as satiety) might persist. A technology assessment report[50] assumed that exenatide would be used instead of insulin for, on average, five years. This is based on its 1% effect on HbA1c, and the expected rise over time of about 0.2% a year due to declining beta cell function. However, data are lacking on whether the underlying decline would be slowed by GLP-1 agonists.

The main interest, or hope, in some of the studies has been whether the GLP-1 agonists might preserve or ever foster recovery of beta-cell function. If they did, this would be a major breakthrough in what is currently a progressive disease. As reported above, in short-term trials the effect on beta-cell function is lost after the GLP-1 agonist is stopped. However, a recent report, available in abstract only, suggests that after three years of exenatide, the beta-cell effect may persist[61].

If GLP-1 analogues do enhance beta-cell survival and stimulate beta-cell growth, they may provide a means to preserve or restore functional beta-cell mass in individuals with type 2 diabetes. More details of the effect on beta-cell function are available in a good recent review by Vilsboll, in which she concluded that longer trials were necessary[62].

### Where do GLP-1 agonists fit into the clinical pathway?

The disadvantages of GLP-1 agonists, compared to other options in dual therapy, are the need to inject, the cost, and the nausea. It does not seem appropriate to start an expensive injectable agent before inexpensive oral drugs have been tried. So the place for the GLP-1 agonists is likely to be in triple therapy, in conjunction with two oral drugs. Their advantage, compared to sulphonylureas and glitazones, is weight loss. This advantage is less in comparison with the gliptins, which are weight neutral.

In patients whose control is inadequate on two oral drugs (usually metformin and a sulphonylurea), the options are: a third oral drug (such as a gliptin or a glitazone); a GLP-1 agonist; or starting insulin. There are no trials of GLP-1 agonists against gliptins or glitazones in this triple therapy situation. There are only two fully-published trials of GLP-1 agonists against a gliptin, and those are in dual combination therapy[38]. Liraglutide appeared more effective than sitagliptin as second drug. However, in routine care, one would try an inexpensive oral drug before an expensive injectable; so the key question is the extent to which liraglutide would improve HbA1c in patients in whom a gliptin had been tried and had failed. In that situation the comparator would be insulin, and following the 4T study[63], the first choice of insulin would be a once daily basal.

In the UK, the NICE guideline suggests that the GLP-1 analogues (at the time, only exenatide was available) should be used as third line agents[3]. NICE also suggest "stopping rules" [3,64] which include a reduction in HbA1c of at least 1%, and 3% weight loss by six months. However, there are different patterns of weight loss. In a pooled post-hoc analysis, Kendall and colleagues reported that some patients treated with exenatide lost weight quickly (in the first 3 months), while others lost weight more slowly over a longer period, but both groups ending up with about 5% body weight loss by two years. About 20% did not lose weight, and while this group also achieved initial improvement in HbA1c, that was rising again in the second year[46]. Similarly, Klonoff and colleagues[65] reported that weight loss continued to 156 weeks, in a pooled follow-up of 415 of patients from three exenatide trials (who may have overlapped with those in the Kendall paper)[46].

The trials against insulin showed weight gain with insulin and weight loss with exenatide and liraglutide. Previous studies have noted that in routine care (as opposed to in trials), treatment with insulin often fails to achieve good control[6], especially in the most overweight[66]. It may be that the benefits of GLP-1 agonists, compared to starting insulin immediately, will be relatively greater as BMI increases.

### On-going trials

There is a need for trials of GLP-1 agonists against insulin in patients who do not achieve adequate control on triple oral therapy, and a need for more trials of combination therapy, with both a GLP-1 agonist and basal insulin, which seems a logical combination in view of their modes of action.

A large number of trials are in progress or planned, involving not only the four GLP-1 agonists described in this review, but newer ones such as lixisenatide and LY2189265. The trials include:

• Head to head comparisons of individual GLP-1 agonists, such as long-acting (once weekly) exenatide versus liraglutide and of weekly taspoglutide versus twice daily exenatide (which may become an obsolescent comparison once long-acting exenatide is licensed).

• Trials of longer intervals between doses, such as a trial of exenatide given once a month.

• Comparisons of GLP-1 agonists versus long-acting insulins.

• Comparisons of GLP-1 agonists with gliptins and glitazones.

• Trials of combination therapy of GLP-1 agonists with insulins.

## Conclusions

GLP-1 agonists are effective in improving glycaemic control when added to dual therapy, and at least in the short term, can be an alternative to starting insulin. The incidence of hypoglycaemia is low, because of their glucose dependent action. They also cause weight reduction, in contrast to the weight gain seen with sulphonylureas, the glitazones and insulin, and the weight neutral effects of the gliptins. How long they would work for in a progressive disease is not yet known. They are a useful addition to the therapeutic armamentarium in type 2 diabetes.

## Competing interests

The authors declare that they have no competing interests.

## Authors' contributions

All authors contributed equally to this work.

All authors have read and approved the final manuscript.

Literature searches: PR; Study selection: DS, PR, PS, NW; Data extraction: CC, DS, PS, PR; Data checking: CC, PR, DS; Data synthesis: DS, CC, PS, PR; Data summary: DS, CC, PR, PS; Preparation of report: DS, PR, NW, CC, PS.

## Pre-publication history

The pre-publication history for this paper can be accessed here:

http://www.biomedcentral.com/1472-6823/10/20/prepub

## Supplementary Material

Additional file 1 **Fasting plasma glucose, post prandial plasma glucose and blood pressure**. Click here for file

Additional file 2 **Adverse events, withdrawals and hypoglycaemia**. Click here for file
