# Supplementary material for: Glucagon-like peptide analogues for type 2 diabetes mellitus: systematic review and meta-analysis
Source: BMC Endocr Disord. 2010 Dec 9;10:20. doi: 10.1186/1472-6823-10-20 (PMC3017518; doi:10.1186/1472-6823-10-20)
Supplement: Additional file 1 — Fasting plasma glucose, post prandial plasma glucose and blood pressure. [file 1472-6823-10-20-S1.PDF]

Table S1. Fasting plasma glucose, post prandial plasma glucose and blood pressure.

Study	FPG (Change from baseline) mmol/L	p value (between groups)	PPG (Change from baseline) mmol/L	p value (between groups)	SBP (Change from baseline) mm Hg	p value (between groups)	DBP (Change from baseline) mm Hg	p value (between groups)
**EXENATIDE**								
Apovian 2010[43]	**Exe:** -2.01 SD2.74<br>**P:** -0.94 SD2.77	p=0.0015	6-point SMBG profile: reduced in both groups	Not given	**Exe:** -9.44 SD13.72<br>**P:** -1.97 SD13.86	p<0.001	**Exe:** -2.22 SD9.8<br>**P:** +0.47 SD9.8	p=0.04
Bergenstal 2009[28]	**Exe:** -1.19 SD3.67<br>**BIAsp BID:** -3.48 SD3.56<br>**BIAsp QD:** -2.91 SD0.32	**Exe vs. BIAsp BID:** p<0.0001<br>**Exe vs. BIAsp QD:** p=0.0002	8-point glucose profile: significantly greater reduction at all points with BIAsp BID compared to exenatide; significantly greater reduction at all points except 2 hr after lunch and breakfast with BIAsp QD.	Not given	Not given	Not given	Not given	Not given
Bergenstal 2010[37]	**Exe QW:** -1.8 SD2.9<br>**Sita:** -0.9 SD2.62<br>**Pio:** -1.5 SD2.62	**Exe QW vs. sita:** p=0.0038<br>**Exe QW vs. pio:** p=NS	6-point SMBG profile: reduction with exenatide significantly greater than with sitagliptin but not pioglitazone	**Exe QW vs. sita:** p<0.05<br>**Exe QW vs pio:** p=NS	Not given	**Exe QW vs. sita:**p=0.0055<br>**Exe QW vs. pio:** p=NS	The paper says '*change in DBP at week 26 did not differ significantly between groups'*.	Not given
Bunck 2009[29]	**Exe:** -1.6 SD1.8<br>**Glar:** -2.9 SD2.3	p<0.001	7-point SMBG profile: significantly greater reductions in postprandial glucose excursions in exenatide-treated patients than insulin-treated patients	Not given	Not given	Not given	Not given	Not given
Davies 2009[30]	**Exe:** -2.29 SD2.64<br>**Glar:** -3.41 SD2.85	p<0.001	Not given	Not given	**Exe:** -2.9 SD12.98<br>**Glar:** +0.7 SD12.81	p=0.034	**Exe:** -0.5 SD7.57<br>**Glar:** +0.9 SD7.47	p=NS
Davis 2007[31]	Not given	Not given	5-point SMBG profile: significantly better post-prandial control with exenatide after breakfast than insulin; no consistent differences for the other time points	p<0.05	Not given	Not given	Not given	Not given
DeFronzo 2005[42]	**Exe:** -0.6 SD2.13<br>**P:** +0.8 SD2.13		Meal tolerance test:<br>**Exe:** 7.2 SD3.2 mmol/L<br>**P:** 12.8 SD4.5	p<0.05	Not given	Not given	Not given	Not given
DeFronzo 2010[22]	**Exe:** -1.46 SD1.68<br>**Rosi:** -1.80 SD1.68	p=NS	Standard meal tolerance test: AUC for glucose showed significant decrease in all groups	p=0.065	Not given	Not given	Not given	Not given
Derosa 2010[27]	**Exe:** -1.50<br>**Glib:** -1.78	p=NS	**Exe:** -1.67<br>**Glib:** -2.06	p=NS	Not given	Not given	Not given	Not given
Diamant 2010[34]	**Exe QW:** -2.1 SE0.2<br>**Glar:** -2.8 SE0.2	p=0.0001	8-point SMBG profile: both treatments reduced postprandial glucose at all eight time points. **Glargine:** lower glucose concentrations at 0300 h and before breakfast than exe; **Exe:** lower glucose concentrations after dinner than glargine.<br>Exe caused greater reduction in postprandial glucose excursions than did insulin glargine after morning and evening meals.	p<0.0001 (for all eight time points)	**Exe QW:** -3 SE1.0<br>**Glar:** -1 SE1.0	Not given	**Exe QW:** -1 SE1.0<br>**Glar:** -1 SE1.0	Not given
Drucker 2008[41]	**Exe:** -1.4 SD2.4<br>**Exe QW:** -2.3 SD2.4	p<0.0001	**Exe:** -6.9 SD6.06<br>**Exe QW:** -5.3 SD6.08	p=0.0124	**Exe:** -3.4 SD13.34<br>**Exe QW:** -4.7 SD13.38		**Exe:** -1.7 SD8.49<br>**Exe QW:** -1.7 SD8.52	

Study	FPG (Change from baseline) mmol/L	p value (between groups)	PPG (Change from baseline) mmol/L	p value (between groups)	SBP (Change from baseline) mm Hg	p value (between groups)	DBP (Change from baseline) mm Hg	p value (between groups)
Gao 2009[45]	**Exe:** -1.3 SD2.73<br>**P:** -0.2 SD3.1		M-Value (SMBG variability)<br>**Exe:**19.7 SD27.53<br>**P:** 43.8 SD27.42;<br><br>Mean daily glucose (SMBG profile)<br>**Exe:** -2.3 SD3.06<br>**P:** -0.4 SD1.52	p<0.001	Not given	Not given	Not given	Not given
Gill 2010[26]	Not given	Not given	Not given	p=NS	Not given	p=NS	Not given	p=NS
Heine 2005[32]	**Exe:** -1.4 SD2.7<br>**Glar:** -2.9 SD2.9	p<0.001	7-points SMBG profile: mean daily monitored glucose levels were not different between treatment groups; but patients receiving glargine had lower BG at fasting, before meals, and at 3 am , but higher BG after morning and evening meals than patients receiving exenatide [i.e. BG levels were more level with exenatide];<br>AUC for PPG (SMBG profile):<br>**Exe:** -8.9<br>**Glar:** -1;<br><br>Meal test (positive incremental AUC at 4 hr):<br>**Exe:** -8.6<br>**Glar:** -1.3	7-point: fasting, before meals, at 3 pm: p<0.001; morning and evening: p<0.001;<br><br>AUC for PPG: p<0.001;<br><br>Meal test: p<0.05	Not given	Not given	Not given	Not given
Kadowaki 2009[20]	**Exe:** -1.61 SD2.01<br>**P:** +0.33 SD1.71	p<0.001	Not given	Not given	Not given	Not given	Not given	Not given
Kendall 2005[21]	**Exe:** -0.6 SD3.1<br>**P:** +0.8 SD3.14		PPG geometric mean AUC 15 to 180 mins:<br>**Exe:** -474 SE87 mmol/min/L<br>**P:** -3 SE72 mmol/min/L;<br><br>Standardized meal tolerance test:<br>**Exe:** -2.9 SD11.80<br>**P:** +0.0 SD6.29	PPG geometric mean: p<0.001<br><br>Meal tolerance test: p<0.01	Not given	Not given	Not given	Not given
Nauck 2007[33]	**Exe:** -1.8 SD3.18<br>**BIAsp BID:** -1.7 SD3.15		7-point SMBG profile:<br>**Exe:** significantly lower mean BG at 2 h post-breakfast and post-supper;<br>**BIAsp:** significantly lower mean BG at pre-breakfast, pre-lunch and 3 am than exenatide	Post-breakfast and post-supper: p<0.001; Pre-breakfast, pre-lunch, 3pm: p<0.05	**Exe:** -5.0 SD15.0<br>**BIAsp BID:** +1.0 SD16.0		**Exe:** -2.0 SD10.0<br>**BIAsp BID:** +1.0 SD10.0	
Zinman 2007[67]	**Exe:** -1.59 SD2.42<br>**P:** +0.1 SD2.22		SMBG:<br>**Exe:** significantly lower than baseline in exenatide group,<br>**P:** no change compared to baseline	p<0.001	Not given	Not given	Not given	Not given

Study	FPG (Change from baseline) mmol/L	p value (between groups)	PPG (Change from baseline) mmol/L	p value (between groups)	SBP (Change from baseline) mm Hg	p value (between groups)	DBP (Change from baseline) mm Hg	p value (between groups)
**LIRAGLUTIDE**								
Kaku 2010[18]	**Lir 0.6mg:** -2.3<br>**Lir 0.9mg:** -2.28<br>**P:** -0.64	**Lir grp vs. P:** p<0.0001	7-point SMBG profile:<br>**Lir 0.6mg:** -2.66<br>**Lir 0.9mg:** -2.89<br>**P:** -0.35	**Lir grp vs P:** p<0.0001	SBP did not change in both groups (Data not given)	p=NS	DBP did not change in both groups (Data not given)	p=NS
Marre 2009 (LEAD 1)[39]	**Lir 1.2mg:** -1.57<br>**Lir 1.8mg:** -1.59<br>**P:** +1.01<br>**Rosi:** -0.88	**Lir grp vs. Rosi:** p≤0.006	Average of values obtained 90 min after breakfast, lunch and evening meal:<br>**Lir 1.2mg:** -2.5<br>**Lir 1.8mg:** -2.7<br>**P:** -0.4<br>**Rosi:** -1.8	**Lir 1.2 vs. Rosi:** p=0.043;<br>**Lir 1.8 vs. Rosi:** p=0.0022;<br>**Lir grp vs. P:** p<0.0001	**Lir 1.2:** -2.6 to -2.8<br>**Lir 1.8:** -2.6 to -2.8<br>**P:** 0.9 to 2.3<br>**Rosi:** 0.9 to 2.3	**Lir grp vs. P:** p=NS;<br>**Lir grp vs. Rosi:** p=NS	**Lir 1.2:** -0.7 to -1.4<br>**Lir 1.8:** -0.7 to 1.4<br>**P:** -0.7 to -1.4<br>**Rosi:** -0.7 to -1.4	**Lir grp vs. P:** p=NS;<br>**Lir grp vs. Rosi:** p=NS
Nauck 2009 (LEAD 2)[36]	**Lir 1.2:** -1.6<br>**Lir 1.8:** -1.7<br>**P:** +0.4<br>**Glim:** -1.3	**Lir grp vs. P:** p<0.0001;<br>**Lir vs. Glim:** p=NS	7-point SMBG profile:<br>**Lir 1.2:** -2.3<br>**Lir 1.8:** -2.6<br>**P:** -0.6<br>**Glim:** -2.5	**Lir grp vs. P:** p<0.0001	**Lir 1.2:** -2 to- 3<br>**Lir 1.8:** -2 to -3<br>**P:** +0.4<br>**Glim:** -1.8	**Lir 1.2 vs. Glim:** p=0.0128;<br>**Lir 1.8 vs. Glim:** p=0.04	DBP did not change from baseline in any groups (Data not given)	p=NS
Zinman 2009 (LEAD 4)[44]	**Lir 1.2:** -2.2<br>**Lir 1.8:** -2.4<br>**P:** -0.4	**Lir grp vs. P:** p<0.0001	7-point SMBG profile:<br>**Lir 1.2:** -2.6<br>**Lir 1.8:** -2.7<br>**P:** -0.8	**Lir grp vs. P:** p<0.001	**Lir 1.2:** -6.7 SD14.68<br>**Lir 1.8:** -5.6 SD14.68<br>**P:** -1.1 SD15.96	**Lir grp vs. P:** p<0.05	**Lir 1.2:** -2.3 SD9.34<br>**Lir 1.8:** -1.9 SD9.34<br>**P:** -0.8 SD9.31	Not given
Russell Jones 2009 (LEAD 5)[35]	**Lir 1.8:** -1.55<br>**P:** +0.53<br>**Glar:** -1.79	**Lir v.s P:** p<0.0001	7-point SMBG profile:<br>**Lir 1.8:** -1.81<br>**P:** +0.03<br>**Glar:** -1.61	**Lir vs. P:** p<0.0001;<br>**Lir vs. Glar:** p=NS	**Lir 1.8:** -4<br>**P:** -1.4<br>**Glar:** -0.54	**Lir vs. P:** p=0.0791;<br>**Lir vs. Glar:** p=0.0001	Non significant reduction in DBP (Data not given)	p=NS
Buse 2009 (LEAD 6)[40]	**Lir 1.8:** -1.61 SD3.05<br>**Exe:** -0.6 SD3.04	**Lir vs. Exe:** p<0.0001	Breakfast: ETD 1.33, 0.80 to 1.86,; Dinner: 1.01, 0.44 to 1.57	Breakfast: p<0.0001;<br>Dinner: , p=0.0005;<br>Lunch: p=NS	**Lir 1.8:** -2.51 SD17.55<br>**Exe:** -2.00 SD17.93	**Lir vs. Exe:** p=NS	**Lir 1.8:** -1.05 SD10.84<br>**Exe:** -1.98 SD 10.79	**Lir vs. Exe:** p=NS
Pratley 2010[38]	**Lir 1.2:** -1.87 SD2.23<br>**Lir 1.8:** -2.14 SD2.21<br>**Sita:** -0.83 SD2.22	**Lir grp vs. Sita:** p<0.0001	Reported that 'PPG was highly variable suggesting that glucose values were not PPG in many cases'. (Data not given)	Not given	**Lir 1.2:** -0.55 SD13.23<br>**Lir 1.8:** -0.72 SD13.14<br>**Sita:** -0.94 SD13.17	**Lir grp vs. Sita:** p=NS	**Lir 1.2:** -0.71<br>**Lir 1.8:** +0.07<br>**Sita:** -1.78	**Lir 1.2 vs. Sita:** p=NS;<br>**LIR 1.8 vs. SITA:** p=0.0210;<br>**LIR 1.8 vs. 1.2:** p=NS
**ALBIGLUTIDE**								
Rosenstock 2009[23]	**Albi 30 QW:** -1.44 SD2.03<br>**Albi 30 every two weeks:** -1.58 SD2.06<br>**P:** -0.10 SD2.90	**Albi QW vs. P:** p<0.05;<br>**Albi two weeks vs. P:** p<0.05	Not given	Not given	**Albi 30 QW:** -5.8 SD11.2<br>**Albi 30 every two weeks:** -7.4 SD14.2<br>**P:** -0.7 SD13.9	**Albi QW vs. P:** p=NS;<br>**Albi two weeks vs. P:** p=NS	**Albi 30 QW:** -1.9 SD8.1<br>**Albi 30 every two weeks:** -4.4 SD8.9<br>**P:** -1 SD8.2	**Albi QW vs. P:** p=NS;<br>**Albi two weeks vs. P:** p=NS
**TASPOGLUTIDE**								

Study	FPG (Change from baseline) mmol/L	p value (between groups)	PPG (Change from baseline) mmol/L	p value (between groups)	SBP (Change from baseline) mm Hg	p value (between groups)	DBP (Change from baseline) mm Hg	p value (between groups)
Ratner 2010[25]	**Tas 20 QW:** -2.3 SD1.7<br>**P:** -0.6 SD1.7	p<0.0001	Not given	Not given	Not given	Not given	Not given	Not given
Nauck 2009[24]	**Tas 10 QW:** -2.5<br>**Tas 20 QW:** -2.5<br>**Tas 20 every two weeks:** -1.4<br>**P:** -0.78	Not given	plasma glucose 120 min after a mixed meal (% change from baseline): TAS 10 QW: -22%<br>**Tas 20 QW:** -18%<br>**Tas 20 every two weeks:** -5.5%<br>P: -10.5%;<br><br>Glucose AUC (from baseline):<br>**Tas 10 QW:** -27.5%<br>**Tas 20 QW:** -22.2%<br>**Tas 20 every two weeks:** -9.2%<br>**P:** -7.2%;	Not given	Not given	Not given	Not given	Not given

FPG: Fasting Plasma glucose; PPG: Postprandial glucose; SBP: Systolic blood pressure; DBP: Diastolic Blood pressure; BG; Blood glucose; Exe: Exenatide; Lir: Liraglutide; Albi: Albiglutide; Tas: Taspoglutide; BIAsp: Biphasic insulin aspart; Glar: Insulin glargine; Glib: Glibenclamide; Glim: Glimepiride; Sita: Sitagliptin; Rosi: Rosiglitazone; P: Placebo; BID: twice daily; QD: Once daily; QW: once weekly; SMBG: Self monitored blood glucose; AUC: Area under the curve; SD: Standard deviation; SE: Standard error; NS: Not significant; LEAD: Liraglutide Effect and Action in Diabetes
