# Supplementary material for: Glucagon-like peptide analogues for type 2 diabetes mellitus: systematic review and meta-analysis
Source: BMC Endocr Disord. 2010 Dec 9;10:20. doi: 10.1186/1472-6823-10-20 (PMC3017518; doi:10.1186/1472-6823-10-20)
Supplement: Additional file 2 — Adverse events, withdrawals and hypoglycaemia. [file 1472-6823-10-20-S2.PDF]

Table S2 Adverse events, withdrawals and hypoglycaemia.

Study	Withdrawal due to adverse events, n (%)	Overall adverse events, n (%)	Gastro intestinal events, n (%)				Serious adverse events, n (%)	Pancreatitis, n (%)	Positive immunogeneity	Deaths, n (%)	Mild/moderate/overall hypos n (%)	Severe hypos n (%)
			**Nausea**	**Vomiting**	**Diarrhoea**	**Other GI events**						
**EXENATIDE**												
Apovian 2010 (Exe/P)[43]	4 (4.2)/5 (5.1) NS	Not given	43 (44.8)/ 19 (19.4) P<0.001	21 (22)/ 9(9) P=0.017	Not given	Not given	2 (2)/2 (2)	Not given	Not given	***No deaths occurred***	*Overall rate (events/pt/year):* 7.1/4.6 (NS); *with Met:* 0.3/0.8 (NS); *with Met + Su:* 13.4/4.7 (p=0.039); *with Su:* 13.5/6.6 (p=0.046); *Nocturnal (events/pt/yr):*1.76/0.95 (NS)	None in all groups
Bergenstal 2009 (Exe/BIAsp 30 BID/QD)[28]	9 (7.30) /6(4.8)/ 1 (0.8)	9 (7.3)/1(0.8)/ 6 (4.8)	***Any GI events:*** 36 (29)/10 (8)/11 (9) ***Nausea/vomiting:*** 7 (5.6)/1 (0.8)/0 (0)				**BIAsp 30 BID:** 2(1.6) (hyperglycaemia and hypokalaemia) possibly related to drug	Not given	Not given	**BIAsp 30 BID:** 1 (0.8) (not related to drug)	Combined hypo episodes (major, minor,sympotoms only) 36 (29)/76 (61)/69 (56);	0(0)/6 (4.8)/4 (3.2)
Bergenstal 2010 (Exe QW/Sita/Pio)[37]	11 (6.9)/5 (3)/ 6 (3.6)		38(24)/ 16 (10)/ 8 (5)	18 (11)/ 4 (2)/ 5 (3)	29 (18)/ 16 (10)/ 12 (7)	*9 (6)/ 3 (2)/ 2 (1)*	4 (3)/ 5 (3)/10 (6)	0/0/2 (1)	Low: 74 (48); Not detectable: 61 (40)	**Sita:** 1 fatal (uncontrolled hypertension)	Minor: 2 (1)/5(3)/1(1); no. of minor events: 2/9/1	None in all groups
Bunck 2009 (Exe/Glar)[29]	5 (13.9)/0 (0)	Not given	18 (50)/Not given	More frequent with **Exe**	More frequent with **Exe**	*Abdominal distension:* More frequent with **Exe**; *Influenza and gastritis:* more frequent with **Glar**	Not given	**Exe:** 1 (resolved after withdrawal of medication)	Not given	Not given	Overall: 3 (8.3)/ 8 (24.2)	None in all groups
Davies 2009 (Exe/Glar)[30]	7 (5.9)/4 (3.4)	No. with at least one TRAE 106 (89.8)/81 (94) NS	57 (48.3)/3 (2.6)	Not given	22 (18.6)/14 (12.1)	Not given	5 (4.2)/5 (4.3)	Not given	Not given	Not given	All episodes 59 (50)/68 (59.6) (p=0.139)	5 (4.2)/ 6 (5.3) (p=0.716)
Davis 2007 (Exe/Ins)[31]	5 (15)/0 (0)	26 (79)/9 (56)	16 (48.5)/2 (12.5)	Not given	Not given	Not given	**Exe:** 2 in 1 patient (chest pain and hyperglycaemi	Not given	Not given	Not given	Overall 13 (39)/6 (38); *Overall rate (events/pt/yr):* 1.72/0.97	3 episodes in 1 patient taking **Exe** and **Su**);

Study	Withdraw al due to adverse events, n (%)	Overall adverse events, n (%)	Gastro intestinal events, n (%)				Serious adverse events, n (%)	Pancreatiti s, n (%)	Positive immunoge neity	Deaths, n (%)	Mild/moderate/over all hypos n (%)	Severe hypos n (%)
			Nausea	Vomitin g	Diarrhoe a	Other GI events						
							a)					None in all other groups
DeFronzo 2005 (Exe/P)[42]	8 (7.1)/1 (0.9)	Not given	51 (45)/26 (23)	13 (12)/4 (4)	18 (16)/9 (8)	Not given	3 (2.7)/4 (3.5)	Not given	43%, no effect on HbA1c;	Not given	Overall: 6 (5.3)/6 (5.3)	None in all groups
DeFronzo 2010 (Exe/Rosi)[22]	2 (4)/1 (2)	Not given	21 (47)/2 (4)	12 (22)/0 (0)	3 (7)/2 (4)	Not given	Not given	Not given	Not given	Not given	Overall: 2 (4)/0 (0)	None in all groups
Derosa 2010 (Exe/Glib)[27]	4 (6.3)/8 (12.3)	Not given	Not given	Not given	Not given	Not given	Not given	Not given	Not given	Not given	Details not given; mentioned that 3 patients taking glibenclamide withdrew because of hypoglycaemia	
Diamant 2010 (Exe QW/Glar) [34]	11 (4.7)/ 2 (0.9)	162 (70)/136 (61)	30(13)/ 3 (1)	10 (4)/ 3 (1)	20 (9)/ 8 (4)	*Constipation: 7(3)/ 4 (2)* *Gastroenteritis: 5 (2)/ 4 (2)*	11 (5)/10 (4)	1 (0.4)/0	87/127 (68%); no effect on HbA1c and safety	***No deaths occurred***	Minor: 19 (8)/58 (26) No. of minor events: 46/135 Symptoms only (no. of events): 73/298	Taking + Met: 1 (0.4)/1 (0.4); Taking Met + SU: 0/1(0.4)
Drucker 2008 (Exe/Exe lar)[41]	7 (4.8)/9 (6.1)	10% or more of patients who received one or more doses had adverse events	50 (34.5)/ 39 (26.4)	27 (18.6)/ 16 (10.8)	19 (13.1)/20 (13.5)	*Constipation:* 9 (6.2)/16 (10.8)	5 (3.4)/8 (5.4); none related to study drug	***No cases of pancreatitis***	More antibodies with **Exe lar** (p=0·0002) but mostly not detectable	Not given	Overall: 9 (6.1)/8 (5.4)	None in all groups
Gao 2009 (Exe/P)[45]	23 (9.8)/3 (1.3)	134 (57.3)/84 (36.2)	59 (25.2)/2 (0.9)	37 (15.8)/ 0 (0)	9 (3.8)/6 (2.6)	*Abdominal distension:* 6 (2.6)/4 (1.7); *Anorexia:* 9 (3.8)/1 (0.4) *Dyspepsia:* 8 (3.4)/1 (0.4)	3 (1)/4 (2)	Not given	34%; no effect on HbA1c and safety	***No deaths occurred***	83 (35.5)/21 (9.1) (p<0.001); *Overall rate (events/pt/yr):* 4.4/0.5	2 patients (both on Su)/1 patient (not on Su)
Gill 2010 (Exe/P)[26]	Not given	Not given	10 (36)/5 (19)	Not given	Not given	Not given	Not given	Not given	Not given	Not given	Minor: 2 (7)/1 (4)	None in all groups
Heine 2005 (Exe/Glar)[32]	27 (9.6)/2 (0.7)	Overall incidence of TEAE occurred	161 (57.1)/ 23 (8.6) (p<0.00	49 (17.4)/ 10 (3.7) (p<0.00	24 (8.5)/8 (3.0) (p=0.006)	*Constipation /(p=0.011) /dyspepsia/(p=0.01 1) anorexia*	Not given	Not given	43%; no effect on HbA1c and safety	Not given	*Overall (events/pt/yr):* 7.3/6.3; *Nocturnal (events/pt/yr):*	4 (1.4)/4 (1.5) (all resolved with oral

Study	Withdrawal due to adverse events, n (%)	Overall adverse events, n (%)	Gastro intestinal events, n (%)				Serious adverse events, n (%)	Pancreatitis, n (%)	Positive immunogeneity	Deaths, n (%)	Mild/moderate/overall hypos n (%)	Severe hypos n (%)
			Nausea	Vomiting	Diarrhoea	Other GI events						
		in at least 2% of treated patients.	1)	1)		*(p=0.002):* 10 (3.5) each/ 1 (<1) each					0.9/2.4; *Daytime (events/pt/yr):* 6.6/ 2.7	carbohydrates)
Kadowaki 2009 (Exe/P)[20]	6 (16.2)/1 (2.5)	35 (94.6)/26 (65.0)	13 (35.1)/0 (0) (p<0.001)	3 (8.1)/ 0 (0) (p=0.067)	3 (8.1)/0 (0) (p=0.044)	*Anorexia (p=0.002) /decreased appetite (p=0.003):* 5 (13.5)/0 (0)	0 (0)/0 (0)	0 (0)/0 (0)	51.4%; no effect on HbA1c and safety	***No deaths occurred***	Mild: 20 (54.1)/4 (10)	None in all groups
Kendall 2005 (Exe/P)[21]	22 (9.1)/11 (4.5)	Not given	117 (48.5)/ 51 (20.6)	33 (13.7)/ 11 (4.5)	42 (17.4)/16 (6.5)	Not given	12 (5)/15 (6)	Not given	49%; no effect on HbA1c and safety	Not given	Overall: 67 (27.8)/31 (12.6)	None in all groups
Nauck 2007 (Exe/BIAsp 30 BID)[33]	20 (7.9)/0 (0)	179 (70.8)/ 123 (49.6)	84 (33.2)/1 (0.4)	38 (15.0)/ 8 (3.2)	24 (9.5)/5 (2.0)	*Dyspepsia/anorexia:* 7 (2.8) each/1 (<1)	19 (7.5)/11 (4.4)	Not given	45%; no effect on HbA1c and safety	2 (0.8)/1 (0.4)	*Overall (events/pt/yr):* 4.7/5.6;(NS) *Nocturnal (events/pt/yr* (NS)): 0.6/1.1; *Daytime (events/pt/yr):* 4.1/4.4 (NS)	None in all groups
Zinman 2007 (Exe/P)[67]	19 (15.7)/2 (1.8)	92 (76.0)/73 (65.2)	48 (39.7)/ 17 (15.2) (p<0.05)	16 (13.2)/ 1 (0.9) (p<0.05)	7 (5.8)/3 (2.7) (NS)	Dyspepsia: 9 (7.4)/1 (0.9) (p<0.05)	2 (1.7)/ 0 (0)	Not given	40%; no effect on HbA1c and safety	Not given	Overall 13 (10.7)/ 8 (7.1) (NS)	None in all groups
**LIRAGLUTIDE**												
Kaku 2010 (Lir 0.6/Lir 0.9/P)[18]	3 (3)/2 (2)/2 (2)	67 (76.1)/69 (78.4)/66 (75)	More subjects in the two liraglutide groups reported gastrointestinal adverse events during the first 4 weeks of the trial than subjects on placebo, but there were no major differences in gastrointestinal adverse events across groups.				3 (3)/2 (2)/2 (2)	***No cases of pancreatitis***	Not given	***No deaths occurred***	*Minor (events/pt/yr):* 2.17/1.96/1.01	None in all groups
LEAD 1, Marre 2009 (Lir 1.2/Lir 1.8/Rosi/P)[39]	11 (5)/ 9 (4)/ 7 (3)/ 6 (5)	Not given	24 (10.5) (lowest)/ Not given/ Not given/2 (1.8)	**Lir 1.2:** 10 (4.4); Others: Not given	**Lir 1.2:** 18 (7.9); **Others:** Not given		9 (4)/12 (5)/7 (3)/ 3 (3)	1 case of chronic pancreatitis taking 0.6 liraglutide	9-13%, no effect on HbA1c and safety	***No deaths occurred***	Minor: 21 (9.2)/19 (8.1)/ 10(4.3)/3 (2.6)	**Lir 1.8:** 1 patient (8 days after treatment started); Not given for other groups

Study	Withdrawal due to adverse events, n (%)	Overall adverse events, n (%)	Gastro intestinal events, n (%)				Serious adverse events, n (%)	Pancreatitis, n (%)	Positive immunogeneity	Deaths, n (%)	Mild/moderate/overall hypos n (%)	Severe hypos n (%)
			**Nausea**	**Vomiting**	**Diarrhoea**	**Other GI events**						
			(lowest)									
LEAD 2, Nauck 2009 (Lir 1.2/Lir 1.8/Glim/P)[36]	23 (10)/29 (12)/8 (3)/2 (2)	Not given	39 (16)/46 (19)/7 to 10 (3 to 4)/4 to 5 (3 to 4)	12 to 17 (5 to 7)/12 to 17 (5 to 7)/3 (1)/1 (1)	19 (8)/36 (15)/10 (4)/ 6 (4)	Not given	**Lir 1.2:** 1 (0.4) (withdraw due to acute pancreatitis)	**Lir 1.2:** 1 (0.4); **Glim:** 1 (0.8)	Not given	2 deaths unrelated to study drug	Minor: 7 (3)/7 (3)/41 (17)/3 (3)	None in all groups
LEAD 4, Zinman 2009 (Lir 1.2/Lir 1.8/P)[44]	11 (6)/27 (15)/6 (3)	Not given	52 (29)/71 (40)/Not given	12 (7)/30 (17)/Not given	Not given	Not given	8 events in 8 patients/ 10 events in 7 patients/13 events in 12 patients	***No cases of pancreatitis***	4.1%; no effect on HbA1c and safety/ 6.7%; no effect on HbA1c and safety	***No deaths occurred***	Minor: 16 (9)/14 (7.9)/9 (5.1)	None in all groups
LEAD 5, Russell Jones 2009 (Lir 1.8/Glar/P)[35]	11 (5);/5 (2.2)/0.90 (1)	Not given	32 (13.9)/ 3 (1.3)/ 4 (3.5)	15 (6.5)/0.4 (1)/ 4 (3.5)	23 (10)/3 (1.3)/6 (5.3)	*Dyspepsia:* 15 (6.5)/4 (1.7)/1 (0.9)	9 (4)/16 (7);/8 (7)	***No cases of pancreatitis***	9.8%; no effect on HbA1c and safety	Not given	Minor: 63 (27.4)/67 (28.9)/19 (16.7)	**Lir 1.8:** 5 (2.2); None in all other groups
LEAD 6, Buse 2009 (Lir 1.8/Exe)[40]	23 (10)/31 (13)	175 (74.9)/182 (78.9)	59 (25.5)/ 65 (28.0)	14 (6.0)/23 (9.9)	29(12.3)/28 (12.1)	*Dyspepsia:* 21 (8.9)/11 (4.7) *Constipation:* 12 (5.1)/6 (2.6)	12 (5.1)/6 (2.6); Only one event related to study medication Severe: 17 (7.2)/11 (4.7)	**Lir:** 1 (0.4) (chronic and unrelated to drug)	Not given	Not given	Minor: 60 (25.5)/78 (33.6)	None/2 episodes
Pratley 2010 (Lir 1.2/Lir 1.8/Sita)[38]	14 (6.3)/15 (6.9)/4 (1.8)	146 (66)/159 (73)/127 (58)	46 (21)/59 (27)/10 (5)	17 (8)/21 (10)/9 (4)	16 (7)/25 (11)/10 (5)	10 (5)/11 (5)/6 (3)	6 (3)/6 (3)/4 (2)	***No cases of pancreatitis***	Not given	0 (0)/1 (<1)/1 (<1); unrelated to study drug	Minor: 12 (5)/11 (5)/10 (5)	**Lir 1.2:** 1 (0.45); None in all other groups
**ALBIGLUTIDE**												
Rosenstock 2009 (30 QW/ every two weeks/P)[23]	5 (16)/5 (15.6)/6 (11.8)	26 (83.9)/27 (84.4)/34 (66.7)	8 (25.8)/8 (25)/6 (11.8)	4 (12.9)/3 (9.4)/1 (2)	5 (16.1)/7 (21.9)/2 (3.9)	*Abdominal pain:* 0 (0)/1 (3.1)/1 (2)	Not given	***No cases of pancreatitis***	2 of 31 (6.4)/1 of 32 (3.1);/1 of 51 (2)	Not given	Overall: 0 (0)/2 (3.9)/2 (3.9)	None in all groups
**TASPOGLUTIDE**												
Ratner 2010 (20 QW/P)[25]	0 (0)/1 (3.1)	Not given	12 (38)/4	4 (13)/0 (0)	4 (13)/3 (9)	*Dyspepsia:* 6 (19)/0 (0)	None/1 (3.1) (unrelated to	Not given	Not given	Not given	Overall: 1 (3)/1 (3)	None in all groups

Study	Withdrawal due to adverse events, n (%)	Overall adverse events, n (%)	Gastro intestinal events, n (%)				Serious adverse events, n (%)	Pancreatitis, n (%)	Positive immunogeneity	Deaths, n (%)	Mild/moderate/overall hypos n (%)	Severe hypos n (%)
			Nausea	Vomiting	Diarrhoea	Other GI events						
			(13)			*Abdominal distension:* 3 (9)/0 (0)	treatment drug)					
Nauck 2009 (10 QW/20 QW/ 20 every 2 weeks/P)[24]	2 (4.1)/3 (6)/1 (2)/0 (0)	Not given	12 (24)/26 (52)/20 (41)/3 (6)	2 (4)/11 (22)/12 (24)/2 (4)	5 (10)/5 (10)/9 (18)/4 (8)	*Dyspepsia*: 4 (8)/6 (12)/2 (4)/ Not given *Abdominal distension:* 2 (4)/2 (4)/6 (12)/ Not given	6 patients; **Tas:** 4 (unrelated to the drug); **P:** 2	Not given	Not given	Not given	no data reported for the separate comparison groups; overall, there were 7 hypoglycaemic events in 6 patients, 2 of which were asymptomatic; there were no cases of severe hypoglycaemia in the taspoglutide group	

Exe: Exenatide; Exe lar: Long acting exenatide; Lir: Liraglutide; Tas: Taspoglutide; Met: Metformin; Su: Sulphonylurea; BIAsp: Biphasic insulin aspart; Glar: Insulin glargine; Ins: Insulin; P: Placebo; Sita: Sitagliptin; Pio: Pioglitazone; Rosi: Rosiglitazone; Glib: Glibenclamide; Glim: Glimepiride; GI: Gastrointestinal; QD: Once daily; BID: Twice daily; QW: Once weekly; HbA1c: Glycated haemoglobin; TRAE: Treatment related adverse events; TEAE: Treatment emergent adverse events; NS: Not significant; events/pt/yr: events per patient per year; hypos: hypoglycaemias
